# Adaptive genomic structural variation in the grape powdery mildew pathogen, *Erysiphe necator*

**DOI:** 10.1186/1471-2164-15-1081

**Published:** 2014-12-09

**Authors:** Laura Jones, Summaira Riaz, Abraham Morales-Cruz, Katherine CH Amrine, Brianna McGuire, W Douglas Gubler, M Andrew Walker, Dario Cantu

**Affiliations:** Department of Viticulture and Enology, University of California Davis, One Shields Ave, Davis, CA 95616 USA; Department of Plant Pathology, University of California Davis, One Shields Ave, Davis, CA 95616 USA

**Keywords:** Fungal genomics, Copy number variation, Genetic adaptation, Fungicide resistance, Cytochrome p450, CYP51

## Abstract

**Background:**

Powdery mildew, caused by the obligate biotrophic fungus *Erysiphe necator*, is an economically important disease of grapevines worldwide. Large quantities of fungicides are used for its control, accelerating the incidence of fungicide-resistance. Copy number variations (CNVs) are unbalanced changes in the structure of the genome that have been associated with complex traits. In addition to providing the first description of the large and highly repetitive genome of *E. necator*, this study describes the impact of genomic structural variation on fungicide resistance in *Erysiphe necator*.

**Results:**

A shotgun approach was applied to sequence and assemble the genome of five *E. necator* isolates, and RNA-seq and comparative genomics were used to predict and annotate protein-coding genes. Our results show that the *E. necator* genome is exceptionally large and repetitive and suggest that transposable elements are responsible for genome expansion. Frequent structural variations were found between isolates and included copy number variation in *EnCYP51*, the target of the commonly used sterol demethylase inhibitor (DMI) fungicides. A panel of 89 additional *E. necator* isolates collected from diverse vineyard sites was screened for copy number variation in the *EnCYP51* gene and for presence/absence of a point mutation (Y136F) known to result in higher fungicide tolerance. We show that an increase in *EnCYP51* copy number is significantly more likely to be detected in isolates collected from fungicide-treated vineyards. Increased *EnCYP51* copy numbers were detected with the Y136F allele, suggesting that an increase in copy number becomes advantageous only after the fungicide-tolerant allele is acquired. We also show that *EnCYP51* copy number influences expression in a gene-dose dependent manner and correlates with fungal growth in the presence of a DMI fungicide.

**Conclusions:**

Taken together our results show that CNV can be adaptive in the development of resistance to fungicides by providing increasing quantitative protection in a gene-dosage dependent manner. The results of this work not only demonstrate the effectiveness of using genomics to dissect complex traits in organisms with very limited molecular information, but also may have broader implications for understanding genomic dynamics in response to strong selective pressure in other pathogens with similar genome architectures.

**Electronic supplementary material:**

The online version of this article (doi:10.1186/1471-2164-15-1081) contains supplementary material, which is available to authorized users.

## Background

**Figure 1:**
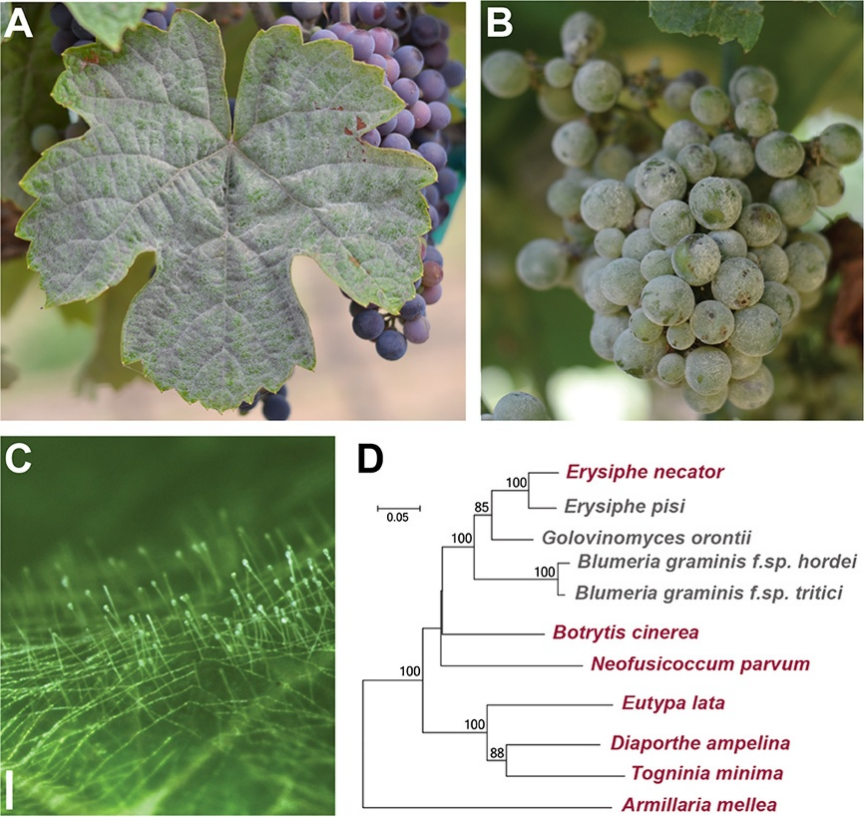
**Powdery mildew disease symptoms and phylogenetic relation of** ***E. necator*** **with other grapevine fungal pathogens and other powdery mildew pathogens.** *E. necator* infections are initiated when conidia come in contact with a susceptible host and germinate, forming hyphae with multilobed appressoria and penetration pegs. Haustoria are formed within the epidermal cell membrane to maintain the parasitic relationship with the host. Young colonies are macroscopically visible and appear white on the surface of **(A)** leaves, **(B)** fruit, and other green tissue. Multiseptate conidophores form along the hyphae perpendicularly to the epidermis, and **(C)** conidia begin to develop within a few days of the initial infection (white bar = 0.2 mm). **(D)** Phylogenetic relationship of *E. necator* with other powdery mildews (gray) and grape fungal pathogens (red). The Neighbor-Joining tree was constructed in MEGA5 [4] using the complete rDNA ITS (ITS1, 5.8 rDNA, ITS2). Multiple publicly available ITS sequences per species were used independently to confirm clustering. The percentage of replicate trees in which the associated taxa clustered together in the bootstrap test (1,000 replicates) is shown next to the branches [5]. Only bootstrap values greater than 60 are shown. The evolutionary distances were computed using the Maximum Composite Likelihood method [6] and are in the units of the number of base substitutions per site. The analysis involved 11 nucleotide sequences. All positions containing gaps and missing data were eliminated. There were a total of 372 positions in the final dataset.

Grapevine powdery mildew is one of the most widespread and devastating diseases of wine, table and raisin grapes, the vast majority of which are cultivars of *Vitis vinifera*. This disease is caused by the fungus *Erysiphe necator* Schw. [syn. *Uncinula necator* (Schw.) Burr.], an obligate biotroph that can infect all green tissues of a grapevine (Figure 1A-C [1]). Infected leaves exhibit reduced photosynthesis and often undergo premature senescence and abscission. Early berry infection causes berries to crack, and the overall impact on the crop includes decreased yields, increased acidity, and decreased anthocyanin and sugar content of mature fruit [2]. Even low levels of powdery mildew infection on the berries can lead to ruined table grapes and wines with negative sensory attributes and decreased varietal character [2, 3].

Most cultivated varieties are susceptible to powdery mildew, and as a consequence growers are forced to apply fungicides to control the disease, often as frequently as every 7–10 days when disease pressure is high. It has been estimated that as much as 20% of the total costs associated with wine grape production in California goes to expenses related to powdery mildew control [7]. Elemental sulfur was the first effective fungicide recommended for vineyards in 1848 to control powdery mildew, and it continues to be widely used, mainly due to its efficacy and low cost [8]. Although its multi-site mode of action remains effective at controlling powdery mildew, the limitations to sulfur use include phytotoxicity at higher temperatures, the need for application as a protectant at frequent intervals, potential off-characters in wine, and the risk of unintended environmental consequences [9, 10]. Since the 1960s, new classes of fungicides have been developed and introduced, many with single-site modes of action and beneficial properties such as systemic effectiveness and longer times between applications [8]. One important class of single-site fungicides is the sterol demethylase inhibitors (DMI), which includes the azole fungicides. DMIs inhibit fungal growth by targeting the cytochrome P450 lanosterol C-14α-demethylase (CYP51, also known as ERG11). CYP51 is a key enzyme in eukaryotic sterol biosynthesis, catalyzing the removal of the 14-α carbon of lanosterol during biosynthesis of ergosterol, the major fungal sterol and an important membrane component [11]. Ergosterol depletion and accumulation of deleterious methylated sterols caused by CYP51 inhibition affect membrane integrity and functionality, reducing fungal growth [12].

DMIs are prone to resistance issues due to their single metabolic target. Triadimefon was the first DMI fungicide to be approved for powdery mildew control in vineyards in 1982, and resistance was documented as soon as 1986 [9, 13]. Resistance to other DMIs has since been reported, including cross-resistance between different compounds [14]. DMI resistance in fungi has been associated with several molecular mechanisms, such as *CYP51* mutations [11] and increased expression of *CYP51* [15]. A single point mutation in *CYP51* causing a tyrosine for phenylalanine substitution at position 136 (Y136F) is associated with DMI resistance in *E. necator* [16], and similar mutations conferring DMI resistance have been identified in other fungi including *Blumeria graminis* f. sp. *hordei* (*Bgh*, henceforth [17]), *Candida albicans* [18], and *Mycosphaerella graminicola* [19]. The amino acid 136 is within the highly conserved CR2 domain involved in substrate recognition [20]. Structural modeling has demonstrated that DMIs bind CYP51 at the sixth ligand of the heme group, and it has been suggested that the Y136F mutation confers resistance by obstructing the heme-binding site [21].

Pathogen populations, particularly species with mixed sexual and asexual reproduction systems like *E. necator*, rapidly evolve to circumvent host resistance genes or to counteract chemical control methods [22]. Though new fungicides are being developed and breeding for genetic resistance is ongoing, little is known regarding how *E. necator* evolves to become resistant to fungicides or how it overcomes host immune responses. Whole-genome sequencing, transcriptomics, and comparative genomic approaches have been successfully applied to other fungal plant pathogens [23]. However, publicly available genomic information for *E. necator* is scarce and limited to SSR markers [24–26], likely reflecting the difficulty of working with an obligate parasite. Genomic analyses of related powdery mildew species (Figure 1D), including the ascomycetes that cause powdery mildew in barley (*Bgh*; [27, 28]) and wheat (*B. graminis* f.sp. *tritici*; *Bgt*, henceforth; [29]) have provided insights into their evolution and mechanisms of pathogenicity.

In this study we applied a shotgun approach to sequence the genomes of five isolates of *E. necator*, and used RNA-seq and comparative genomics to predict and annotate protein-coding genes. Our results not only show that the *E. necator* genome is exceptionally large and repetitive compared to other fungal plant pathogens (as previously seen in other powdery mildews), but also that frequent structural variations occur between field isolates. Structural variation resulted in a wide range of copy numbers in the DMI fungicide target gene *CYP51* (*EnCYP51*). A panel of 89 additional *E. necator* isolates collected from both synthetic fungicide-treated and non-treated vineyard sites was screened for both copy number variation and presence/absence of the Y136F mutation in the *EnCYP51* gene. The vast majority (95%) of isolates with increased copy number were collected from fungicide-treated vineyards. Increased *EnCYP51* copy numbers were almost always (94%) detected with the *EnCYP51* mutant allele (Y136F), suggesting that an increase in copy number becomes advantageous only when the *EnCYP51* gene is present in its fungicide-tolerant allelic form. We also show that *EnCYP51* genomic copy number is correlated with both its mRNA transcript level and with fungal growth in the presence of DMI fungicide*.* Taken together, our results demonstrate that copy number variation can be adaptive in the development of resistance to DMI fungicides in *E. necator*.

## Results

### Genome sequencing and assembly of five *E. necator*isolates

Between 2012 and 2013, 94 *E. necator* isolates were collected from seven vineyard sites in California (Additional file 1: Table S1). Five isolates with diverse genetic and geographical backgrounds (Branching, C-strain, e1-101, Lodi, and Ranch9) were selected for genome sequencing based on microsatellite profiling results, collection site, and fungicide treatment history (Additional file 1: Table S1; Additional file 2: Figure S1; Additional file 3: Text S1). The Branching and e1-101 isolates were collected from vineyard sites without fungicide treatment, while the C-strain, Lodi, and Ranch9 isolates were all collected from sites regularly treated with synthetic fungicides to control powdery mildew.

We used an Illumina shotgun sequencing approach to sequence the haploid genomes of the five selected *E. necator* isolates. We generated both medium reads (∼250 bases) and short reads (∼150 bases) using the Illumina MiSeq and HiSeq2500 sequencers, respectively. The longer reads were used to assemble the draft genome sequence for the C-strain isolate, while the shorter reads were used for resequencing and *de novo* assembly of the other isolates. After quality trimming and filtering, most sequencing reads (98.5 ± 0.2%) were assembled into 6,475 ± 444 scaffolds with a total length of 50.5 ± 1.2 Mb (Table 1; Additional file 4: Table S2; mitochondrial DNA scaffolds: 188 kb ± 1.3 kb). Most of the reads of each isolate (97.4 ± 0.3%) aligned as pair-ends to the assembled contigs of the other isolates suggesting a similar genome completeness and representation across all five isolates. Unambiguously mapped reads were used to assess single nucleotide polymorphism (SNP) frequencies between the isolates following the GATK pipeline for SNP calling. The GATK analysis identified an average SNP frequency of 0.79 ± 0.08 SNPs/kb (Additional file 5: Table S3).

**Table 1:** ***E. necator*** **genome assembly statistics**

Isolate	Median coverage	Number of scaffolds	Total assembly size (Mb) ^1^	N50 scaffold length (kb)	L50 scaffold count	CEGMA complete (%)
C-strain	76X	5,936	52.5	21.4	710	96.8
Lodi	42X	6,884	49.8	14.5	1,011	96.4
Ranch9	24X	6,979	49.5	14.1	1,022	96.4
Branching	29X	6,225	50.7	18.4	813	96.0
e1-101	42X	6,350	50.0	17.2	870	95.6

^1^Genome size estimated by *K-mer* frequency was 126 ± 18 Mb.

Based on the distribution of DNA *k-*mer counts [30], we estimated the genome size of *E. necator* at 126 ± 18 Mb, which is similar to the 120 Mb estimated genome size of *Bgh* [27, 28] and significantly smaller than the 180 Mb genome size estimated for *Bgt* [29]. The discrepancy between assembled scaffold size and estimated genome size suggests that a large proportion of the *E. necator* genome is highly repetitive and collapsed into common contigs during assembly. This hypothesis is also supported by the high sequencing coverage observed in the highly fragmented and gene-poor fraction of the assemblies (Additional file 6: Figure S2A). In contrast, the longest and most gene-rich contigs exhibit the expected sequencing coverage based on estimated genome size and sequencing depth (Additional file 6: Figure S2A).

Depth of coverage was used to estimate the size of the interspersed repeats. Repeats, including transposable elements (TEs), microsatellites, and low-complexity regions, were estimated to represent 62.9 ± 3% of the *E. necator* genome (Additional file 6: Figure S2B), a proportion similar to the repeat content estimated for the *Bgh* genome (64%; [27]). TEs were annotated using a combination of *ab initio* discovery and comparative analysis. TEs accounted for 77.9 ± 3.6% of the total repeats, with LTR retrotransposons and LINEs being the most abundant classes of TEs in the genome (Additional file 6: Figure S2B). TEs were abundant among the sequenced transcripts, suggesting that in *E. necator* TEs are transcriptionally active (Additional file 6: Figure S2D).

To assess the quality and completeness of the assembled gene space, we applied the CEGMA pipeline [31] to map a set of 248 low copy Core Eukaryotic Orthologous groups (KOGs) which are conserved across higher eukaryotes. On average 96.2 ± 0.5% of the KOGs aligned as complete gene copies to the scaffolds, compared to 98.1 ± 0.4% that aligned as fragmented partial gene copies (Table 1; Additional file 4: Table S2). These results indicate that our shotgun sequencing approach generated a nearly complete assembly of the *E. necator* gene space, despite the obvious failure in resolving the structure of the repetitive fraction of the genome. For the analyses described below we used the genome assembly of the C-strain isolate as a reference, because it appeared to be the most complete based on assembly metrics and CEGMA evaluation (Table 1; Additional file 4: Table S2).

### Gene prediction and annotation

To enhance gene discovery and gene structure prediction, we sequenced and assembled the *E. necator* transcripts expressed during the interaction with grape leaves at 0.5, 1, 3, and 6 days after conidia inoculation (Additional file 7: Table S4; Additional file 8: Figure S3A&B). Assembled transcripts were then used as evidence in a gene prediction pipeline that combined evidence-based, homology-based, and *ab initio* methods (Additional file 8: Figure S3C). After removing 4,511 genes associated with TEs, we estimated that the *E. necator* genome contains 6,533 protein-coding genes. A similar total number of protein-coding genes was identified in *Bgh* (6,470 [27]; Additional file 9: Table S5) and *Bgt* (6,525 [29]). Nearly 92% of the predicted genes have detectable similarity to proteins known from other ascomycetes (BLASTP e-value < 10^−3^; NCBI Ascomycete nr; Additional file 9: Table S5; Additional file 10: Table S6; Additional file 11: Figure S4). Percent BLASTP matches and alignment coverage between the peptides of *E. necator* and other fungi, support the overall quality of the gene prediction pipeline results (Additional file 12: Table S7). Prediction accuracy was also supported by the identification of higher frequencies in nucleotide variants in the intergenic space (0.89 ± 0.01 SNPs/kb) and intronic regions (0.57 ± 0.07 SNPs/kb) than in the exons (0.35 ± 0.04 SNPs/kb; Additional file 5: Table S3). An average of 1.7 introns per gene were predicted and, similarly to observations in other fungal genomes [32], intron lengths had a narrow distribution with mean size of 83.7 bp (exon mean size = 526.5 bp; Additional file 8: Figure S3D-F). Genes on longer scaffolds displayed shorter intergenic spaces than those on smaller scaffolds, suggestive of an uneven distribution of genes within the genome (Additional file 8: Figure S3G). As reported in previous analyses of powdery mildews [27, 28] and other obligate biotrophs [33–35], the *E. necator* genome showed a loss of enzymes involved in secondary metabolism, nitrate and sulfate metabolism, further supporting the convergent adaptation of obligate biotrophy (Additional file 3: Text S1 [23]). We did not find any of the genes known to be required for Repeat Induced Point mutation (RIP) suggesting that the mechanisms that usually control TEs in fungi are only partially functional in *E. necator*, as previously observed in other powdery mildew pathogens (Additional file 3: Text S1; [27]).

### Secretome and candidate effector proteins

Microbial plant pathogens secrete effector proteins, some of which have been shown to subvert the plant innate immune response and enable infection [36]. The predicted secretome of *E. necator* contained a total of 607 genes. GO term enrichment analysis of the secretome showed enrichment in hydrolase and peptidase molecular function (MF) terms (Additional file 13: Table S8), although many of these enzymes possess β-1,3-glucanase or chitinase activity and are likely involved in remodeling the pathogen’s own cell wall during early infection instead of breaking down the host tissue as in necrotrophy [37].

The *E. necator* secretome also included several classes of proteins that were previously identified as pathogen candidate effectors, such as EKA-like proteins [38] and ribonucleases-like proteins [39, 40]. These putative effector proteins were detected in transcriptome data from early infection time points suggesting that some might be involved in the very early stages of infection (Additional file 10: Table S6; Additional file 14: Table S9; Additional file 6: Figure S2D). Following the same approach described in [27], we identified 150 candidate secreted effector proteins (CSEPs; Additional file 3: Text S1), which displayed a significant enrichment in sequence motifs associated with secreted peptides of haustoria-forming pathogenic fungi ([41]; Additional file 14: Table S9). These candidate effectors did not present any signature of positive selection unlike those described in other plant pathogens ([42]; Additional file 3: Text S1). The absence of elevated levels of non-synonymous polymorphisms among the candidate effectors may hypothetically reflect the absence of antagonistic evolution in the host, since almost all cultivated grape varieties are fully susceptible to powdery mildew.

### Structural variation between *E. necator*isolates

**Figure 2:**
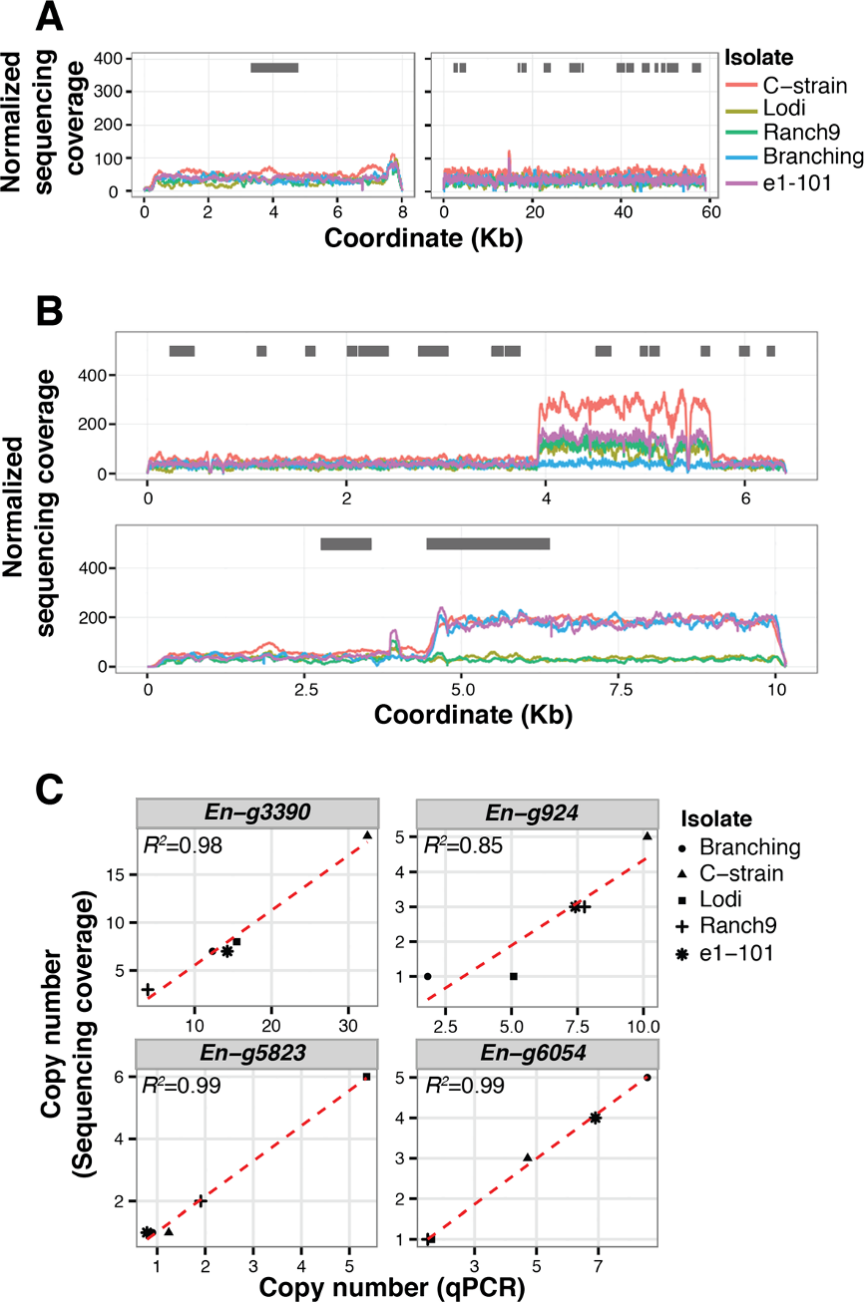
**Structural variation between** ***E. necator*** **isolates estimated by sequencing depth analysis and qPCR.** Line plot showing examples of genomic loci **(A)** without copy number variation (CNV), and **(B)** with CNV across the five genomes. Loci with copy number polymorphisms were detected by analyzing sequence read depth with CNV-seq [45]. Gray boxes depict protein-coding sequences. **(C)** Validation by qPCR of CNV-seq copy number calls for four sample CNV loci in the five sequenced isolates. Scatterplots show the correlation between estimated copy numbers for both methods. A linear trend line is shown. Copy numbers by qPCR were estimated based on 2^−ΔCt^ values normalized to the single copy *E. necator* elongation factor gene *EnEF1* (*En-g1817*).

Copy number variations (CNVs) are unbalanced changes in the structure of the genome and include deletions, insertions, and duplications of >1 kb in size [43]. Although short sequencing read length and limited insert size do not allow reliable characterization of the type of structural variation underlying CNV, variation in sequencing coverage in genome assemblies can be used as an indicator of CNV between an assembled genome and sequencing reads from another genome ([44]; Figure 2A&B). To detect structural variants between the five sequenced isolates, we applied the CNV-seq pipeline to the mapped reads [45]. CNV-seq calculates the read-depth signal across all genomic coordinates using a sliding window and was shown to provide more accurate relative copy number estimation than other CNV-detection tools [46]. Genome-wide CNV-seq results are summarized in Table 2. While the sizes of the detected CNVs were similar across the isolates, the total number of CNV sites had a broad range from 177 to 1,170, corresponding to 1.1% and 5.3% of the assemblies, respectively. The identified CNV loci not only spanned TEs (LTR: 364 ± 32 kb; non-LTR TEs: 99 ± 35 kb; Additional file 6: Figure S2C), but also 135 protein-coding genes (49.3 ± 4.0 CNV genes/genome; Additional file 10: Table S6). A broad diversity of genes was observed within CNV loci with no detectable enrichment for any particular function. CNV-seq estimations of copy number polymorphisms were validated by quantitative PCR (qPCR; Figure 2C).

**Table 2:** **Genome-wide estimates of copy number variation in the five sequenced** ***E. necator*** **isolates determined by CNV-seq analysis (** ***P*** **< 0.001** [45]**)**

Isolate	Copy number total size (bp)	% assemblies	Median size (bp)
e1-101^1^	543,296	1.0	5,408
Ranch9^1^	644,367	1.2	5,137
C-strain^1^	1,030,577	2.0	4,668
Lodi^1^	2,769,800	5.3	5,500
Branching^2^	556,500	1.1	5,040

^1^Values represent CNV-seq results when Branching scaffolds were used as reference.

^2^Values represent CNV-seq results when e-101 scaffolds were used as reference.

### Copy number variation of the *EnCYP51*locus in the five sequenced isolates

**Figure 3:**
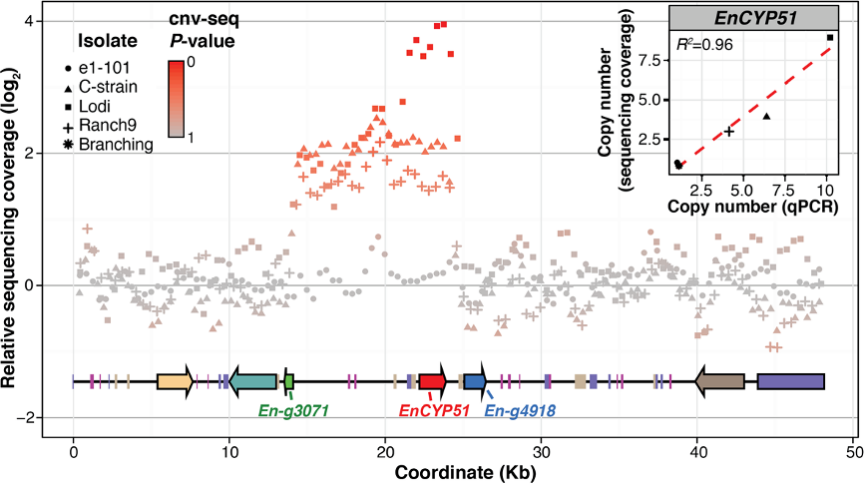
**Copy number variation of the** ***EnCYP51*** **locus.** *Log2* ratio plot of sequencing depth in the *EnCYP51* locus of e1-101, C-strain, Lodi, and Ranch9 relative to the sequence depth in the *EnCYP51* locus of Branching. CNV was confirmed in all pair-wise comparisons using CNV-seq. Depth of coverage of the *EnCYP51* locus in the Branching isolate is presented as it provided the longest assembled scaffold of the locus across all isolates. Arrows and boxes depict protein coding sequences and transposable elements, respectively. Duplication boundaries upstream and downstream of the *EnCYP51* coding region were common in all isolates with multiple copies of *EnCYP51*. Only in Lodi did an additional duplication event occur involving a shorter region of 7.4 kb. CNV-seq estimates of *EnCYP51* copy numbers were validated using qPCR. Scatterplot in inset shows the correlation between CNV-seq and qPCR copy number estimates (red dashed line = linear trend line). In all isolates the duplication events did not involve the two flanking genes, *En-g4918* and *En-g3071*, which were confirmed by qPCR to be single copy in all genomes (Figure 6D).

The *EnCYP51* gene, which encodes the enzyme targeted by DMI fungicides, was among the protein-coding genes with a wide range of copy number variation across the five sequenced genomes. Sequencing coverage, confirmed by qPCR, estimated the presence of 3, 4, and 9 *EnCYP51* copies in Ranch 9, C-strain, and Lodi, respectively, and single copies in e1-101 and Branching (Figure 3). Remarkably, the three isolates collected from fungicide-treated vineyards showed multiple copies of *EnCYP51* with all copies being the Y136F mutated allele, while the two isolates from vineyards not treated with fungicides both had single copies of the *EnCYP51* gene in its fungicide-susceptible allelic form (Additional file 15: Figure S5A&B). The Y136F substitution was the only non-synonymous polymorphism detected between the isolates in the *EnCYP51* coding region (Additional file 15: Figure S5C). All *EnCYP51* alleles were confirmed by Sanger sequencing, which also confirmed that all *EnCYP51* duplicated copies were in the same allelic form (Additional file 15: Figure S5B).

**Figure 4:**
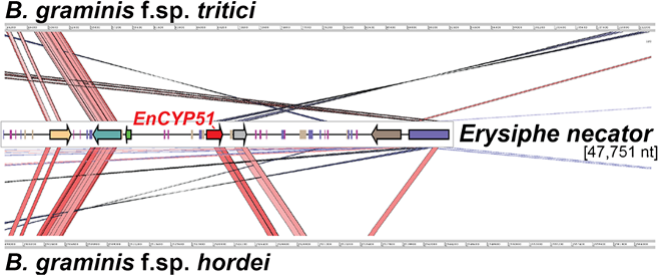
**Microsynteny of the** ***EnCYP51*** **locus between** ***E. necator*** **,** ***Blumeria graminis*** **f.sp.** ***hordei*** **(contig: bgh_dh14_v3.0_contig_002323), and** ***Blumeria graminis*** **f.sp.** ***tritici*** **(scaffold: KE375157.1).** Scaffold alignment based on translated nucleotides (TBLASTX) and similarity visualization was done using ACT-Artemis Comparison Tool (http://www.sanger.ac.uk/resources/software/act/). Red and blue lines indicate similar regions between scaffolds (% similarity ≥ 60). Blue lines indicate inversions. Arrows correspond to protein-coding genes.

Duplication boundaries, approximately 8.0 and 0.6 kb upstream and downstream of the *EnCYP51* coding region, respectively, were common in all isolates (Figure 3). Only in Lodi did an additional duplication event occur involving a shorter region of 7.4 kb (Figure 3). In all isolates, the duplication events did not involve the two flanking genes, *En-g4918* and *En-g3071*, which were confirmed by qPCR to be single copy in all genomes. In all isolates the downstream duplication boundary coincided with a fragment of a Gypsy element (Figure 3). Although numerous other TEs were found throughout the sequence flanking the duplicated region, we did not detect repeated homologous sequences that could provide the substrate for non-allelic homologous recombination and allow us to hypothesize the mechanism underlying the copy number polymorphisms in the locus [47]. Microsynteny of the *EnCYP51* locus between *E. necator*, *Bgh*, and *Bgt* (Figure 4) both confirms the accuracy of the sequence assembly and also shows the structural conservation in this region of the genome across species despite the frequent structural rearrangements observed in the *EnCYP51* locus.

**Figure 5:**
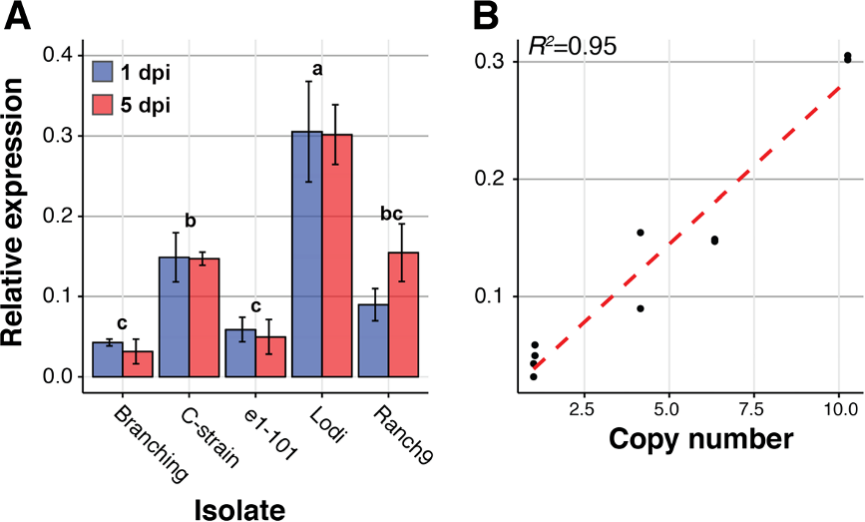
**Impact of CNV on** ***CYP51*** **expression. (A)** Bar graph presenting qRT-PCR data for the relative expression of *EnCYP51* in sequenced isolates at 1 and 5 days post inoculation. Expression values are presented as fold-*EnEF1* (number of *EnCYP51* molecules/number of *EnEF1* molecules), calculated using the ΔCt method as described in [49]. Data were analyzed using a two-way ANOVA. Values are the mean ± standard error of three biological replicates. Different letters represent significant results (Tukey’s HSD test: *P* < 0.01). **(B)** Scatterplot showing the correlation between relative expression of *EnCYP51* by qRT-PCR and copy number predicted by sequencing coverage. A linear trend is shown.

CNVs can modify the expression of genes that localize within the CNV in a gene dose-dependent manner [48]. To determine the impact of CNV on *EnCYP51* expression, we measured mRNA transcript expression levels at 1 and 5 days post conidia inoculation using qRT-PCR. Our results show that *EnCYP51* was differentially expressed in the different isolates at both time points (Figure 5A) with a strong correlation between gene expression levels and *EnCYP51* copy number (Figure 5B).

### Copy number variation of the *EnCYP51*locus occurs frequently in *E. necator*and is correlated with fungal growth under fungicide treatment

**Figure 6:**
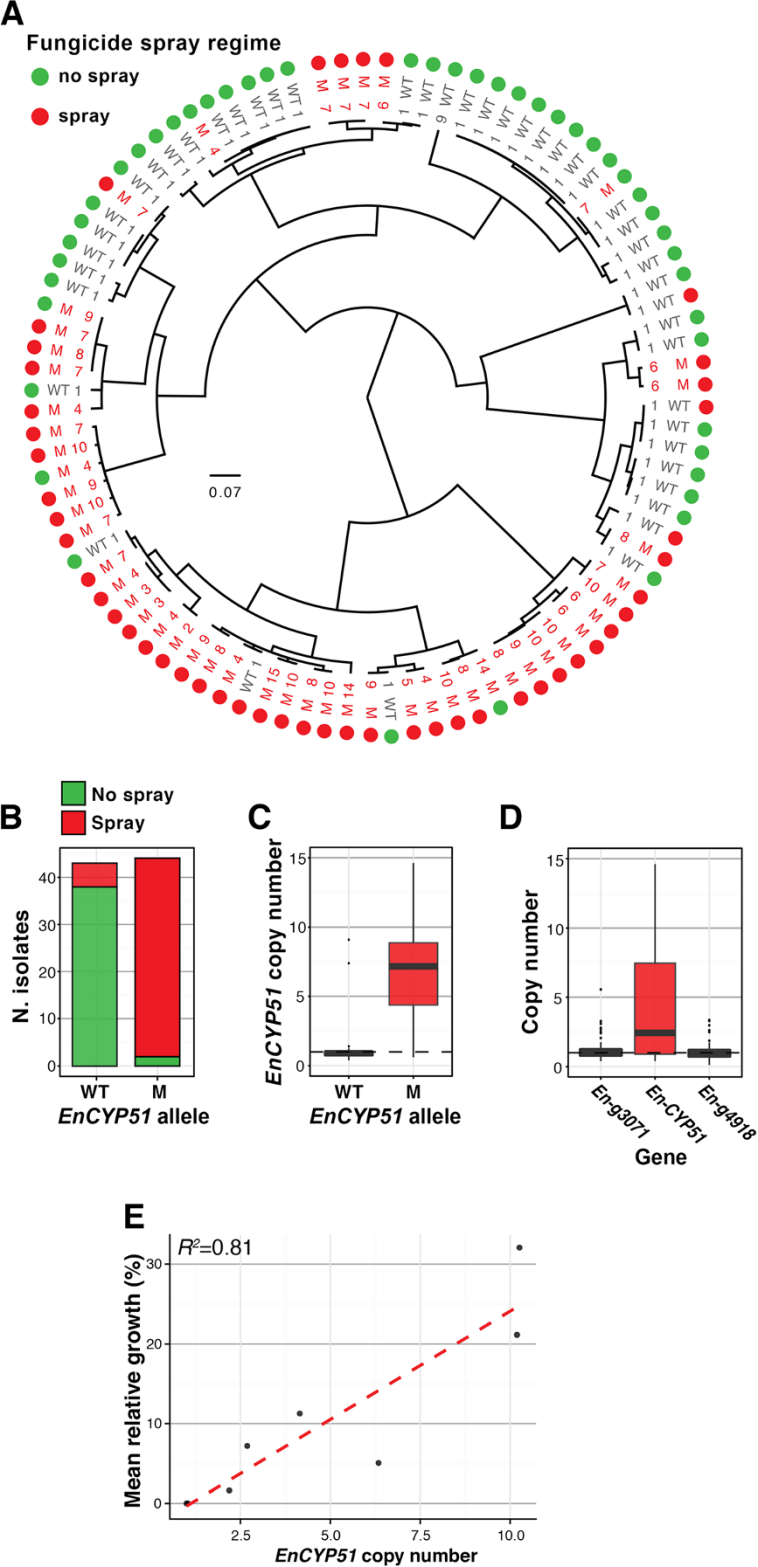
**Copy number variation of the** ***CYP51*** **locus occurs frequently in** ***E. necator*** **and correlates with fungal growth under fungicide treatment. (A)** Circular visualization of the allelic form (M = mutant, WT = wild type) and copy number of *EnCYP51* in the 94 California isolates, and the fungicide spray regime of each collection site. At the center of the circular plot is a hierarchical clustering dendrogram determined by SSR marker-based fingerprinting (Additional file 3: Text S1). **(B)** Bar plots showing the proportion of wild type and mutant *EnCYP51* alleles collected from sites sprayed or not sprayed with fungicides. Box plots showing **(C)** the distribution of *EnCYP51* copy number in isolates containing the mutant allele compared to those with the wild type allele, and **(D)** box plots showing the distribution of copy numbers of the two genes that flank the conserved duplication boundaries observed in the individuals with multiple *EnCYP51* copies. **(E)** Scatterplot shows the correlation between mean relative growth measured by qPCR and copy number estimates. The capability of isolates with different copy numbers of *EnCYP51* to grow in the presence a DMI fungicide (Rally® 40WSP) was measured. To account for growth rate differences among isolates in absence of the fungicide, growth results in presence of the fungicide were calculated as a percentage of the fungal biomass accumulation in absence of the fungicide. Growth values are the mean of two separate assays, each run with three biological replicates.

To determine the extent of *EnCYP51* CNV in *E. necator* beyond the five sequenced isolates, we screened an additional 89 isolates collected from multiple vineyard locations (Additional file 1: Table S1). All isolates tested for CNV were also genotyped to determine the allelic forms of *EnCYP51*. Out of the 89 total isolates, 48% carried the wild-type allele, while 52% carried the Y136F mutation (Figure 6). The majority (88%) of the isolates with the wild type allele were collected from non-sprayed vineyards, while almost all (95%) of the isolates with the Y136F mutation came from sprayed vineyards (Figure 6B). Quantitative PCR analysis showed the presence of multiple copies of *EnCYP51* in 47 isolates with a wide range of copy numbers, from as few as 2 copies and up to 14 copies (Figure 6C). The two genes that flank *EnCYP51* were mostly single copy confirming the conservation of the duplication boundaries (Figure 6D). Remarkably, 94% of the isolates with multiple copies of *EnCYP51* also carried the mutant Y136F allele, while 96% of the isolates with single copies of *EnCYP51* were wild type, further supporting the hypothesis that the acquisition of multiple copies of the fungicide-tolerant allele contributes to the evolutionary fitness. Hypergeometric tests supported the significant association between fungicide treatment and both presence of the Y136F allele (*P =* 4.8 × 10^−4^) and multiple copies of *EnCYP51* (*P = * 4.3 × 10^−5^). The *EnCYP51* CNV and its allelic form did not correlate with SSR-based clustering (Figure 6A; Additional file 3: Text S1) suggesting that the Y136F mutation and the acquisition of multiple of copies of the *EnCYP51* gene occurred multiple times in the *E. necator* populations screened.

To test the hypothesis that an increase in *EnCYP51* copy number leads to stronger resistance to DMI fungicides, we tested the capability of isolates with different copy numbers of *EnCYP51* to grow in the presence of Rally® 40WSP, a DMI fungicide. Eight isolates with a range of CNVs in *EnCYP51* were tested, including the five isolates we sequenced. A qPCR analysis was performed to quantify the accumulation of *E. necator* biomass eight days after conidia inoculation. As expected, under fungicide treatment we did not detect any growth of Branching and e1-101, which are the two isolates that carry the wild type and single copy *EnCYP51*, while a positive correlation between fungal growth in presence of the DMI fungicide and the estimated number of *EnCYP51* copies was observed in the isolates containing the Y136F mutation (Figure 6E; Additional file 16: Figure S6B). While we know the number of *EnCYP51* copies and the *EnCYP51* allelic form in each isolate, we were unable to control for genetic differences in other genes in the genome that may mask or confound the role of *EnCYP51.* For example it has been suggested that ABC transporters contribute to DMI resistance [11], and although we did not see sequence polymorphisms and CNVs that may suggest a contribution of ABC transporters to the differential DMI susceptibility of the isolates we tested, we could not exclude the possibility that other loci in the genome are responsible for the differential growth in response to the fungicide treatment.

## Discussion

This study describes for the first time the genome of one of the most widespread and devastating diseases of grapevines. With an estimated size of ~125 Mb, the *E. necator* genome is among the largest sequenced ascomycete genomes. The *E. necator* genome also possessed a characteristic reduction in protein-coding genes, a feature also seen in the genomes of other biotrophic fungi [50]. The expansion of genome size in powdery mildews [27–29], including *E. necator*, is associated with the proliferation of repetitive DNA, particularly TEs, which account for about 63% of the *E. necator* genome. TEs were also detected in the *E. necator* transcriptome, indicating that they are transcriptionally active at least during host infection*.* Active TEs are a major source of mutations in the genome. Transposition of TEs not only contributes to the expansion of genome size [51], but can also cause chromosome breaks and rearrangements [52], gene deletions [53], gene duplications [54], illegitimate recombination [55], and changes in gene expression [56, 57]. TE activity has also been associated with the evolution of pathogen virulence factors [58].

Frequent structural variations in the genome also point to a highly unstable genomic architecture in *E. necator*, although a direct link between TE proliferation and CNV cannot yet be established based on our results. In this study, the analysis of read depth showed that up to 5% of the genome assemblies, corresponding to approximately 2.8 Mb, are CNV, which indicates that at least among the five isolates we sequenced CNV involves larger genomic regions than DNA sequence polymorphisms (SNP: 41,657 ± 4,151 SNPs/genome). Studies with model organisms, including fungi, plants, and animals, have already shown that the extent of genetic differences between individual organisms due to alterations in the number of gene copies can often be greater than differences in the nucleotide sequences [59]. CNVs can result from different types of structural variations, such as deletions, translocations, inversions, tandem duplications, and novel insertions. Because read depth analysis cannot identify with certainty the type of rearrangement that underlies the CNV, characterization of the type of medium- and large-scale structural genomic variations would require longer reads, larger inserts between paired-end reads, and longer scaffolds than those used in this study. Nonetheless, qPCR validation of CNVs (Figure 2C & Figure 3 inset) confirmed that read depth analysis can be used to reliably detect CNV regions in the genome.

CNVs can influence phenotypic variation and adaptation by modification of gene dosage, gene expression, or disruption of genes that span boundaries of structural rearrangements. CNVs have been associated with phenotypic differences and complex diseases in humans [43], adaptation to different soil types in *Arabidopsis lyrata* [60], higher aluminum tolerance in maize [61], nematode resistance in soybean [62], drug resistance in *Plasmodium falciparum* [63], and DDT resistance in *Drosophila melanogaster* [64] *.* A role of CNV in contributing to effector protein evolution was described for the oomycete plant pathogen, *Phytophtora sojae* [65].

Here we show that (i) the DMI fungicide target *EnCYP51* is CNV in 95% of isolates collected from vineyards treated with fungicides, (ii) increasing *EnCYP51* copy number leads to higher expression of *EnCYP51*, and (iii) increasing *EnCYP51* copy number correlates with increased ability to withstand fungicide treatment. Furthermore, Sanger sequencing confirmed that the vast majority (94%) of the isolates with multiple *CYP51* copies carried the mutant Y136F allele in every gene copy. Our results also show that isolates with identical or very similar SSR haplotypes can have contrasting *EnCYP51* alleles and copy numbers, which suggests that structural rearrangement in the locus and subsequent selection under fungicide pressure happened frequently and on multiple occasions in the populations we screened. This observation is compatible with findings of high CNV rates over family generational scales [66].

While the Y136F mutation reduces the affinity between *CYP51* and the inhibitor [12, 21], sensitivity to DMIs has also been shown to depend on the expression levels of *CYP51*. In other plant pathogens the overexpression of *CYP51* has resulted in decreased susceptibility to DMIs [15, 67–69]. Segmental duplication of a 126-bp fraction of the *CYP51* promoter led to increased expression of CYP51 in a fungicide resistant strain of *Penicillium digitatum* [67], while duplication of the entire chromosome bearing the *CYP51* gene resulted in increased fungicide resistance in the yeast *Candida glabrata* [70]. Although polymorphisms have been found in resistant *CYP51* genes [71, 72], no clear association between a specific mutation and an increase in *CYP51* expression has been reported in fungi.

Based on the strong association between CNV, the Y136F allele, and fungicide treatment, we hypothesize that in *E. necator* CNV can provide an additional layer of genetic diversity that contributes to pathogen fitness once a favorable allele is acquired. The development of increasing quantitative fungicide resistance could happen in two steps. First, occasional *E. necator* individuals with the Y136F mutation in *EnCYP51* survive DMI fungicide treatments and, as a consequence, Y136F allelic frequency increases in vineyards treated with DMIs. Second, structural rearrangements that increase the number of *EnCYP51* copies occur in individuals carrying the Y136F, which are then favored by further DMI treatments. We do not rule out the possibility that structural rearrangements in the *EnCYP51* locus may occur with the same frequency in the presence of the wild type allele, but based on the low frequency of CNV in the wild type allele we observed, the acquisition of multiple copies of the wild type alleles does not seem to provide evolutionary fitness in fungicide treated vineyards. Alternatively, the strong connection between the mutation and multiple copies in *E. necator* could also be a result of a fitness cost due to the Y136F mutation. The mutation occurs in a highly conserved substrate recognition site [20], and perhaps the substitution leads to a less efficient sterol biosynthesis in *E. necator* and the increased copy number is needed to compensate the loss of affinity to the substrate. The compensatory effect provided by the acquisition of multiple CNV copies may explain why out of the 94 isolates we studied none carried the Y136F allele as a single copy gene. However, we did not observe any obvious impact of the Y136F mutation on fungal growth when growth rates of wild type and mutant strains in the absence of fungicide were compared (Additional file 16: Figure S6A).

Taken together, our results show that CNV of the *EnCYP51* gene contributes to *E. necator* fitness by providing increasing quantitative protection against DMI fungicide treatments in a gene-dosage dependent manner. Monitoring powdery mildew evolution for the development of fungicide resistance should include measuring the number of the *EnCYP51* gene copies. This knowledge will help in managing fungicide programs and maintaining effective control of powdery mildew while minimizing the development of populations increasingly resistant to DMI treatments.

## Conclusions

As part of this study we generated valuable genomic information for the grape powdery mildew pathogen, including: (i) whole-shotgun sequence assemblies of five isolates of *E. necator*, (ii) an estimate of the distribution of genetic diversity as both sequence polymorphisms and structural variants among *E. necator* isolates, (iii) characterization of the expression of *E. necator* genes in infected tissues using RNAseq analysis, (iv) gene models of 6,533 protein-coding genes identified with an a hybrid approach that involved both *ab initio* and reference based predictions using genome-guided *de novo* assembled transcripts. In addition to providing the first description of the large and highly repetitive genome, in this work we show that frequent structural variations occur between field isolates, including copy number variation in *EnCYP51*, the target of sterol demethylase inhibitor fungicides. The *EnCYP51* gene in isolates collected from fungicide-treated vineyards contained both a mutation known to result in higher fungicide tolerance and an increased copy number that correlated with gene expression and fungal growth in the presence of fungicides. Our results also suggest that CNV can provide an additional layer of genetic diversity that contributes to pathogen fitness once a favorable allele is acquired.

The results of this work not only demonstrate the effectiveness of using genomics to dissect complex traits in organisms with very limited molecular information, but also may have broader implications for understanding genomic dynamics in response to strong selective pressure in other pathogens with similar genome architectures.

## Methods

### Biological material

A total of 94 *E. necator* isolates were collected between 2012 and 2013 from seven vineyard sites in California. The DNA of four additional isolates from the eastern United States, kindly provided by Dr. L. Cadle-Davidson (USDA-ARS, Geneva, NY), were included in our analysis to allow comparisons of SSR fingerprint data among different studies ([25]; Additional file 1: Table S1). Using the procedure described in [73], isolates were single-spore purified and maintained on detached leaves of *V. vinifera* cv. Carignan. Leaves were surface sterilized for 3 minutes with 3.0% sodium hypochlorite, rinsed with sterile water, dried between layers of sterile paper towels, and placed onto a 0.8% water agar medium in 100 mm × 15 mm Petri dishes with the adaxial side up and the cut petiole inserted into the medium. Four subsequent sub-transfers of a single chain of conidia were made to new leaves to ensure a pure colony was maintained in culture.

### Genome sequencing and assembly

Genomic DNA was extracted from mycelia of each isolate collected from colonies growing on detached Carignan leaves approximately 20 days post inoculation. A cyclone separator tube adapter attached to the vacuum port was used to collect the *E. necator* tissue from the surface of the leaf to minimize plant tissue contamination. DNA was extracted using a modified CTAB protocol [74], and then fragmented using a Diogenode Bioruptor NGS. Sequencing libraries were prepared using the KAPA LTP Library Sequencing Preparation Kit. Insert size selection was carried out using the Life E-gel SizeSelect to achieve a mean library fragment size of approximately 600 bp for the MiSeq and 550 bp for the HiSeq2500. Insert size and library quality was confirmed before sequencing using the Agilent 2100 Bioanalyzer (Agilent Technologies). Sequencing was carried out on Illumina MiSeq and HiSeq2500 machines at the DNA Technologies Core at UC Davis. Sequenced reads were quality trimmed (Q > 20) using sickle (v1.210; [75]) and adapters were removed with scythe (version 0.991; [76]). High quality reads were assembled *de novo* using CLC Workbench v6.1. Assembly parameters were optimized to achieve maximal assembly completeness of the gene space estimated using the Core Eukaryotic Genes Mapping Approach (CEGMA) analysis [31]. All assemblies were generated using a word size of 64 and a bubble size of 244. Contigs with homology only to non-fungal sequences in the complete NCBI nt collection were considered contaminant and discarded. Basic assembly metrics were extracted using the assemblathon_stats.pl script [77] and are shown in Additional file 4: Table S2. Genome sizes were estimated based on *k-*mer count distribution [30].

### Transposable elements annotation

RepeatModeler (v1.0.7; [78]) was used to perform *ab initio* repeat family prediction. The scaffolds of the C-strain isolate were used as the only input for RepeatModeler. With the exception of TEs marked as “unknown”, the repeat sequences identified by RepeatModeler were combined with the RepeatMasker database and used as reference for RepeatMasker (v4.0.2; [79]) to screen the DNA assemblies for interspersed repeats and low complexity DNA sequences.

### Transcriptome sequencing, *de novo*assembly, and gene prediction

Third and fourth fully expanded Carignan leaves were surface sterilized as described above and placed in Petri dishes containing 0.8% water agar medium. A portable paint sprayer was used to inoculate leaves with a 10^6^ conidia/mL suspension of the C-strain isolate in 0.001% Tween. The leaves were maintained under ambient conditions. Time points were collected at 0.5, 1, 3, and 6 days post inoculation. Accumulation of *E. necator* transcripts was confirmed by qRT-PCR (Additional file 8: Figure S3B; Additional file 17: Table S10). For each time point, three independent pools each containing two leaves were collected. RNA was extracted from combined leaf and fungal tissue using a CTAB method [80]. cDNA libraries were prepared using the Illumina TruSeq RNA Sample Preparation Kit. Library quality was confirmed with the Agilent 2100 Bioanalyzer, and sequencing was carried out on the Illumina HiSeq 2500. Sequence reads were quality filtered and trimmed using scythe and sickle as described above. For gene prediction we applied a hybrid approach: (i) transcripts were *de novo* assembled using RNA-seq reads and a genome-guided assembly strategy; (ii) the assembled transcripts were employed both as training set for *ab initio* prediction and as evidence for homology-based prediction together with other fungal proteomes. To identify transcribed loci in the genome for genome-guided transcript assembly, reads were mapped onto the C-strain scaffolds with the spliced (intron-aware) aligner TopHat (version 2.0.9; [81]) with the following parameters: --r 100 --mate-std-dev 50 --min-intron-length 20. Reads mapped on each transcribed partition were then *de novo* assembled independently using Trinity (version r20131110; with jaccard_clip option on [82]). ORFs were extracted using the perl script transcripts_to_best_scoring_ORFs.pl, part of Trinity. *De novo* assembled transcripts together with the gene structures of the CEGs identified in the C-strain genome by CEGMA were used to train Augustus [83] for *ab initio* gene discovery on repeat-masked scaffolds. MAKER (version 2.8; [84]) was then used to integrate the *ab intio* prediction with homology based gene prediction using *Bgh, Bgt*, and *B. cinerea* peptide sequences as templates (Additional file 8: Figure S3C). Predicted peptides matching known TE associated proteins (BLASTP, e-value < 10^−3^) were removed. Predicted transcripts not matching any peptide in NCBI nr (BLASTX, e-value < 10^−3^) were removed unless RNA-seq data mapping provided evidence of their expression. The transcripts of most of these predicted protein-coding genes were present in the RNA-seq data, ranging from 49.1% at 0.5 dpi to 80.6% at 6 dpi of the total 6,533 genes (Additional file 8: Figure S3H-J).

### Gene annotation

Functional annotation was performed using BLASTP (NCBI nr Asomycota database; e-value < 10^−3^, −v 10 -b 10, −F F) followed by Blast2GO [85]. Pfam domains were annotated using the Pfam batch search server ([86]; p-value < 10^−3^), while proteins were assigned to Carbohydrate Active enZYme (CAZy) families based on similarity searches against the non-redundant sequences of the CAZy database using the CAZymes Analysis Toolkit (CAT; [87]). Proteins were clustered in tribes based on sequence similarity using BLASTP (e-value < 10^−6^) followed by Markov clustering with TribeMCL [88]. The presence of secretion signal peptides was evaluated using SignalP v.4.1 [89]. Perl scripts were used to scan the first 110 amino acids of each protein for putative effector motifs as described in [90] (Additional file 10: Table S6).

### Gene expression profiling during an infection time course

The quality filtered and trimmed pair-end RNA-seq reads were aligned to a combined reference of the 6,533 predicted protein-coding transcripts and the *V. vinifera* var. PN40024 genomic scaffolds to profile the *E. necator* genes during susceptible grape leaf infection. Because RNA-seq samples include both *E. necator* and Carignan RNAs, a combined reference was used to minimize the non-specific mapping of *V. vinifera* reads onto *E. necator* transcripts [91]. The aligner Bowtie2 (version 2.1.0; [92]) was used for read mapping with parameters --end-to-end --sensitive. SAM output files were parsed to extract read counts per transcript with the python script sam2counts.py (version 0.91; [93]). Non-specific mappings were discarded by filtering with sam2counts.py (−q 30). DEseq (version 1.12.1, [94]) was used to normalize raw transcript counts and to compare infection time points.

### Assessing genetic diversity

Illumina pair-end genomic sequence reads from each isolate were mapped onto the C-strain scaffold using Bowtie2 (−−end-to-end --sensitive; [92]). Mapping metrics are reported in Additional file 4: Table S2. Non-uniquely mapped reads and optical duplicates were removed with samtools (version 0.1.19; [95]) and Picardtools (version 1.104), respectively. The GATK (version 2.4.3) RealignerTargetCreator and IndelRealigner programs were applied to realign the reads mapped on indel sites. The GATK UnifiedGenotyper was then used to identify SNPs with parameters --glm SNP --min_base_quality_score 20 --ploidy 1 --outputmode EMIT_VARIANT_ONLY. VCF files generated by GATK were then parsed with custom perl scripts to extract counts of intergenic, intronic, and exonic variants. To identify genes that are polymorphic and under positive selection we applied the same procedure as described in [96]. Synthetic sequences incorporating the GATK-detected SNPs were generated using the GATK FastaAlternateReferenceMaker tools. Orthologous transcripts were then aligned using ClustalW and subjected to pair-wise polymorphism and positive selection analysis using the program Yn00 [97]. Any pair-wise comparisons that yielded a dN value > 0 were classified as polymorphic and those with dN/dS values > 1 were classified as under positive selection. SSR fingerprinting was carried out as described in Additional file 3: Text S1 (see also Additional files 18, 19 and 20 for additional information). Missing core-eukaryotic genes in the 5 isolates (Additional file 21: Table S14) were identified as described in Additional file 3: Text S1.

### Estimation of genome-wide copy number variation

Sequence depth of coverage was used to identify CNV loci in the five *E. necator* isolates using CNV-seq [45]. Because CNV-seq requires a control sample for comparison, we performed all possible pair-wise comparisons of the 5 genomes. BAM files filtered to exclude ambiguous mapping and optical duplications as described above were used as input for CNV-seq analysis, which was performed with the parameters --global-normalization --genome-size 120000000 --annotate --p-value 0.001. Gene copy numbers were measured using quantitative PCR. DNA was extracted as described above. Additional file 17: Table S10 lists all qPCR amplified genes and their corresponding primers. qPCR was performed on an Applied Biosystems 7500 PCR System using Power SYBR Green Master Mix. All qRT-PCR reactions were run in triplicates and performed with the following cycling conditions: 50°C for 2 min, 95°C for 10 min, followed by 40 cycles of 95°C for 15 s and 60°C for 60 s. Primer efficiencies were calculated using 4-fold DNA dilutions (1:1, 1:4, 1:16, 1:64, and 1:256) in duplicate. The efficiencies of the primer sets used in this study were all above 90% (Additional file 17: Table S10). Specificity of the primers was checked by analyzing dissociation curves ranging from 60 to 95°C. The 2^−ΔΔ*CT*^ method was used to normalize and calibrate *CYP51* copy numbers as described in [98]. Data were normalized to the *E. necator* single copy elongation factor gene *EnEF1* (*En-g1817*; Figure 2A) by subtracting the *EnEF1* Ct value and using Branching DNA (1 *CYP51* copy) as a calibrator to adjust for any minor variance among the DNA dilution quantities of the different samples as follows: ΔΔ*CT* = (Ct, CYP51 – Ct, *EnEF1*)_x_ – (Ct, CYP51 – Ct, *EnEF1*)_y_, where x = test sample and y = Branching isolate. *CYP51* alleles were determined by PCR as described in [16] and confirmed by Sanger sequencing of PCR amplicons generated using primers listed in Additional file 17: Table S10, and the following PCR conditions: 95°C for 1 min, 46 cycles of 95°C for 15 s, 63°C for 30 s, 68°C for 30 s, and a final extension of 68°C for 7 min.

### Measurement of *CYP51*expression by qRT-PCR

Detached Carignan leaves were inoculated with *E. necator* conidia and RNA was collected 1 and 5 days after inoculation as described above. cDNA was prepared from the isolated RNA using M-MLV Reverse Transcriptase (Promega). All qRT-PCR reactions were performed as follows: 50°C for 2 min, 95°C for 10 min, followed by 40 cycles of 95°C for 15 s and 60°C for 60 s. The *E. necator* elongation factor gene (*En-g1817* or *EnEF1*; Additional file 17: Table S10), whose expression was confirmed to be constant across inoculation time points (Additional file 10: Table S6), was used as the reference gene and processed in parallel with the genes of interest. All expression values are presented as fold-*EnEF1* (number of target molecules/number of EF1 molecules), calculated using the ΔCt method as described in [49].

### Fungicide resistance testing of *E. necator*isolates

Carignan leaves were surface sterilized as described above. A cork borer was used to cut 16 mm diameter leaf disks, and the disks were placed in 6-well tissue culture plates containing 0.8% water agar medium. Rally® 40WSP fungicide (active ingredient: 40% myclobutanil) was applied to the surface of each leaf disk at 7 different concentrations in triplicate with concentrations ranging from 0 to 50 mg/L of the active ingredient. 50 µL of each fungicide dilution was applied to the surface of the leaf disk, and the residual liquid was allowed to evaporate. After 24 hours, the leaf disks were inoculated with one of the five *E. necator* isolates. Each inoculum was prepared as a suspension of 10^5^ conidia/mL in 0.001% Tween, and 30 µL was pipetted onto each leaf disk. The liquid was allowed to evaporate, and the plates were stored at ambient conditions. Eight days post inoculation, combined leaf and fungal DNA was extracted from each disk using the CTAB method and fungal biomass accumulation was determined by qPCR amplification of the *E. necator* elongation factor *EnEF1*. DNA accumulation levels were linearized with the formula 2 ^– (*EnEF1* Ct – *VvACT* Ct)^ using the grape actin gene as reference (Additional file 17: Table S10), which was confirmed not to change with the progression of infection, as reference. To account for growth rate differences among isolates in absence of the fungicide, growth results in the presence of the fungicide were calculated as the percentage of the fungal biomass accumulation in the absence of the fungicide. Relative growth values across all fungicide concentrations are shown in Figure 6E and Additional file 16: Figure S6B.

### Data access

The whole genome shotgun projects have been deposited in the GenBank database [accession nos. JNVN00000000 (isolate C-strain), JNUS00000000 (isolate Branching), JNUT00000000 (isolate Ranch9), JOKO00000000 (isolate e1-101), and JNUU00000000 (isolate Lodi)]. RNA-sequencing data used in this study have been deposited in the National Center for Biotechnology Information Gene Expression Omnibus (GEO) database, http://www.ncbi.nlm.nih.gov/geo (no. GSE58958). The raw reads are available via Sequence Read Archive project no. SRP043708 (accession nos. SRX642773 - SRX642784).

## Electronic supplementary material

Additional file 1: Table S1: *E. necator* isolates used in this study. (XLSX 51 KB)

Additional file 2: Figure S1: Genetic structure of the *E. necator* isolates used in this study. (A) Un-weighted Neighbor-Joining tree constructed with genotypic data from 11 SSR markers with 82 unique haplotypes using DARWIN software. The color-coding of samples is based on the STRUCTURE program clustering from K1 to K4 (see Additional file 18: Table S11). The five isolates that were sequenced are marked by a closed circle. Label colors correspond to the genetic cluster the isolate belongs to based on STRUCTURE analysis. Some admixed samples were grouped together with samples from other populations. (B) Graphical presentation of the estimated membership coefficient, Q, for each of the 82 unique haplotypes in each of four genetic clusters (K). The most likely value of K inferred by STRUCTURE was 4. Each sample is shown as a horizontal line; the colored segments represent the proportion of the Q in each of the four ancestral genetic clusters. Individuals within each cluster are arranged according to the estimated cluster membership proportions (Q value). Detail of accessions in each cluster is provided in Additional file 18: Table S11. (PDF 437 KB)

Additional file 3: Text S1: Supplementary Material and Methods, Supplementary Results, Supplementary References. (PDF 153 KB)

Additional file 4: Table S2: DNA sequencing and genome assembly statistics. (XLSX 47 KB)

Additional file 5: Table S3: SNP density and ratio of transition/transversion (Tr/Tv) nucleotidic changes in intergenic, exonic, and intronic sequences. (XLSX 38 KB)

Additional file 6: Figure S2: Analysis of the repetitive fraction of the *E. necator* genome. **(A)** Scatterplot showing the relation between scaffold length and sequencing coverage. The highly fragmented and gene-poor fraction of the assemblies shows higher sequencing depth than the long and gene-rich contigs, whose coverage match the median (and expected) value (red dashed line; 76X). **(B)** Bar plot showing the proportion of sequencing reads that mapped on the repetitive fraction of the *E. necator* genome. **(C)** Bar plot showing the size of copy number variant loci encompassing the annotated repetitive regions. **(D)** Bar plot showing the total number of RNA-seq reads that mapped on the annotated repetitive regions. (PDF 736 KB)

Additional file 7: Table S4: Time-course RNA-seq sequencing and mapping statistics. (XLSX 51 KB)

Additional file 8: Figure S3: Transcriptome sequencing and gene prediction. **(A)** Proportion of *V. vinifera* and *E. necator* reads in the RNA-seq reads at each time point (hpi = hours post inoculation, dpi = days post inoculation). **(B)** Scatter plot showing the correlation between the expression of *EnCYP51* and β-tubulin gene (*EnBT*) measured by RT-qPCR and the total *E. necator* RNA-seq reads. Expression values are presented as fold-*EnCYP51 or EnBT* (number of *EnCYP51* molecules/number of *VvACTIN* molecules), calculated using the ΔCt method as described in [49]. **(C)** Diagram describing the pipeline used for gene prediction. **(D-F)** Histograms showing size distribution of exons ((D) mean size = 526.5 bp), introns ((E) mean size = 83.7 bp), and intergenic space ((F) mean size = 3,745.6 bp). **(G)** Scatter plot showing the relation between the median size of the intergenic space in each scaffold, scaffold length, and the number of genes per scaffold. Short and gene-poor scaffolds show the largest median intergenic space (top left), while long and gene-rich scaffolds (bottom right) show the smallest median intergenic space. **(H-J)** Bar graphs showing the total number of genes **(H)**, CAZy genes **(I)**, and CSEPs **(J)** mapped by the RNA-seq data during the infection time course. (PDF 3 MB)

Additional file 9: Table S5: Summary of the *E. necator* predicted proteome and comparison with other sequenced fungal genomes and the GenBank nr collection of Ascomycete peptides. (XLSX 41 KB)

Additional file 10: Table S6: Gene Annotation, GenBank locus tags, CNV, and RNA-seq counts. (XLSX 4 MB)

Additional file 11: Figure S4: Characterization of the *E. necator* predicted proteome. **(A)** Venn diagram showing the overlap between predicted peptides with features associated with candidate effectors. Venn diagrams **(B-G)** showing the overlapping *E. necator* predicted peptides **(B)**, CSEPs **(C)**, and CAZymes (D-G; CEs = carbohydrate esterases; CBMs = carbohydrate binding modules; GTs = glycosyltransferases; GHs = glycosylhydrolases) matching the scaffolds of *B. graminis* f.sp. *hordei* (Bgh), *B. graminis* f.sp. *tritici* (Bgt), *G. orontii*, or *E. pisi* (TBLASTN, e-value < 10^−3^). (H) Venn diagram showing the number of unique or overlapping copy number variant protein-coding genes in the five sequenced *E. necator* isolates. (PDF 4 MB)

Additional file 12: Table S7: Result of pairwise reciprocal BLASTP searches. (XLSX 43 KB)

Additional file 13: Table S8: Molecular function GO term enrichment of putative secreted genes. (XLSX 31 KB)

Additional file 14: Table S9: Features of 150 E. *necator* candidate secreted effector (CSEPs). (XLSX 64 KB)

Additional file 15: Figure S5: Presence of the *EnCYP51* mutant allele (Y136F) conferring DMI resistance in isolates collected from fungicide-treated vineyards. The three isolates collected from fungicide-sprayed vineyards (C-strain, Lodi, and Ranch9) showed multiple copies of *EnCYP51* with all copies containing the Y136F mutation, while the two isolates from vineyards not treated with fungicides (e1-101 and Branching) each contained a singly copy of the wild type, fungicide susceptible *EnCYP51* allele. **(A)** Diagram of sequencing read coverage and the presence of Y136F mutation. Boxed solid red line represents the presence of only one allele at the 136 position of *EnCYP51*. Multicolored lines represent positions with mixed alleles in other regions. **(B)** Sanger sequencing chromatogram of *EnCYP51.* Single peaks at the 136 position confirm that all duplicated copies are in the same allelic form. All 94 California isolates were genotyped as described in [16] and allelic form were validated by Sanger sequencing. **(C)** Protein alignment of *EnCYP51* shows that the Y136F substitution was the only non-synonymous polymorphism detected across the isolates. (PDF 2 MB)

Additional file 16: Figure S6: Fungicide resistance testing of *E. necator* isolates. Eight *E. necator* isolates were grown on leaf disks with and without DMI fungicide application (Rally® 40WSP). Results are an average of two separate trials, each with 3 independent replicates. Allelic form (WT = wild type, M = Y136F mutant) and *EnCYP51* copy number estimated by qPCR are indicated in parentheses. **(A)** Box plot showing biomass accumulation in the absence of fungicide determined by qPCR amplification of the *E. necator* elongation factor *EnEF1*. **(B)** Boxplot showing the distribution of relative growth values. DNA accumulation levels were linearized with the formula 2 ^– (*EnEF1* Ct – *VvACT* Ct)^ using the grape actin gene as reference. Growth results are reported as a percentage of the fungal biomass in the absence of fungicide (shown in (A)) to account for growth rate differences between the isolates in the absence of fungicide. (PDF 407 KB)

Additional file 17: Table S10: Primer sequences used in this study. (XLSX 34 KB)

Additional file 18: Table S11: The estimated membership coefficients (Q values) for 82 isolates in each of the four genetic clusters (K) inferred by STRUCTURE. Isolates that were admix with Q-values less than 70% are in light grey color for each cluster. (XLSX 43 KB)

Additional file 19: Table S12: Microsatellite allele frequencies of *E. necator* isolates in this study. Allele sizes are reported in base pairs (bp). Frequency of alleles were calculated for California samples (61 unique haplotypes) and comparisons were made after the addition of four reference samples from the Eastern USA and synthetic data of Type-A and B isolates. Bold and italicized microsatellite alleles were observed only in isolates from California. (XLSX 28 KB)

Additional file 20: Table S13: Microsatellite marker allele data for 94 *E. necator* isolates from California. Four isolates (bold and Italicized) previously used in [25] from the Eastern USA were used as reference in this study to transform data between two study sets. Samples with missing data are shown as ‘ND’. Five isolates denoted with * were used for sequencing. (XLSX 52 KB)

Additional file 21: Table S14: Missing core ascomycete genes in *E. necator* and their presence (+) or absence (−) in the other sequenced powdery mildew pathogens. (XLSX 65 KB)
