# Supplementary figures and images for: Adaptive genomic structural variation in the grape powdery mildew pathogen, Erysiphe necator
Source: BMC Genomics. 2014 Dec 9;15(1):1081. doi: 10.1186/1471-2164-15-1081 (PMC4298948; doi:10.1186/1471-2164-15-1081)

A

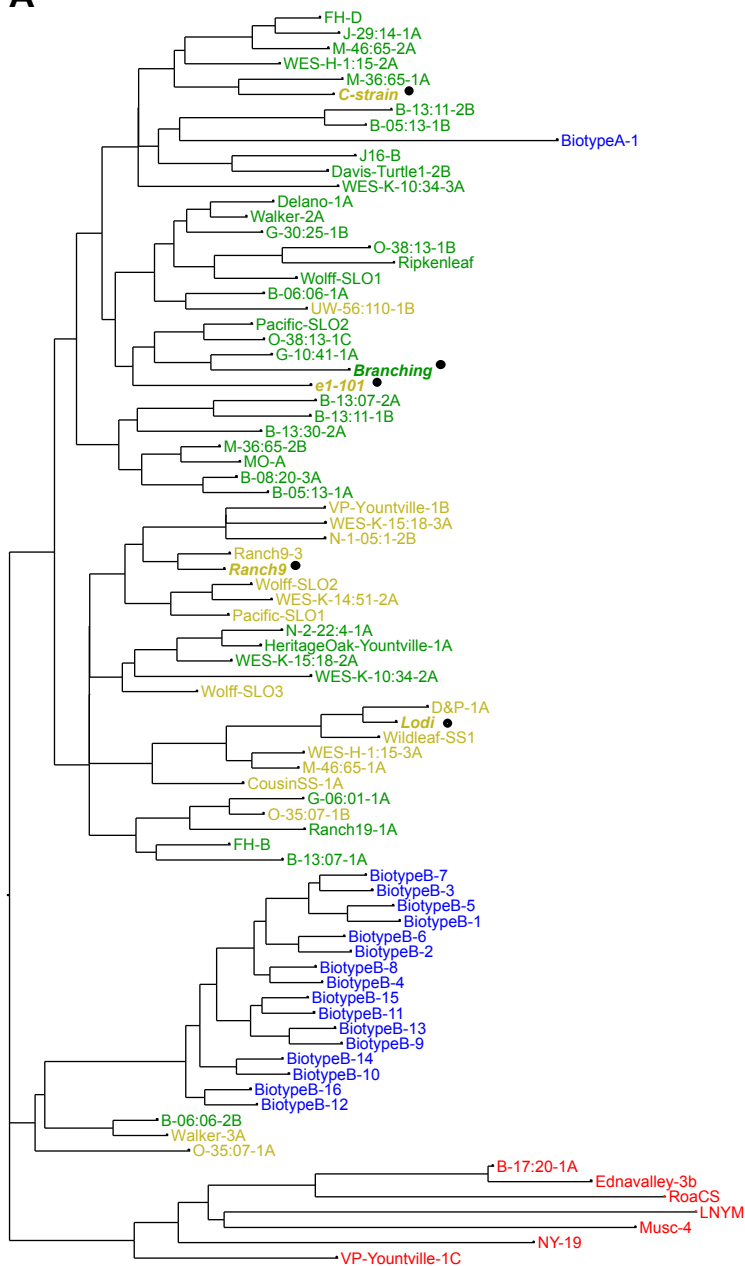

B

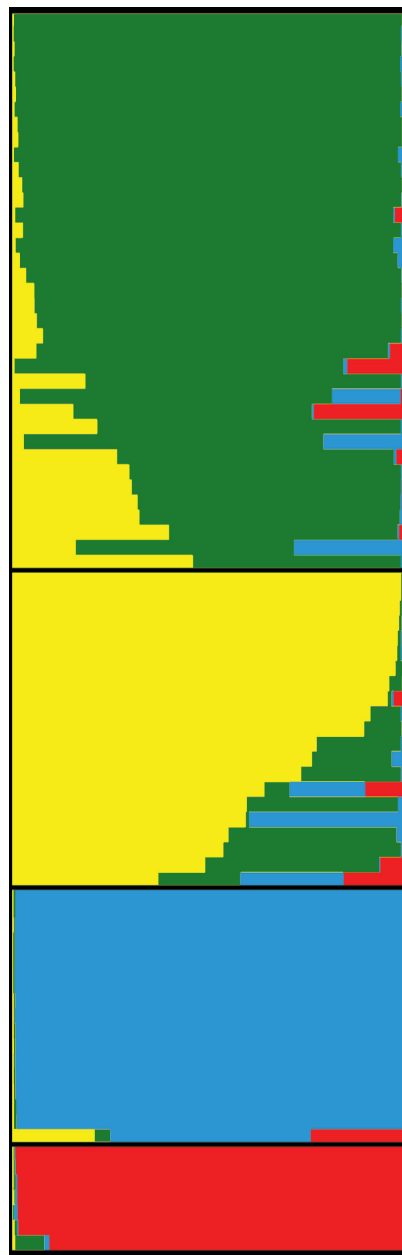

Supplement: Supplementary file 2 — Additional file 2: Figure S1: Genetic structure of the E. necator isolates used in this study. (A) Un-weighted Neighbor-Joining tree constructed with genotypic data from 11 SSR markers with 82 unique haplotypes using DARWIN software. The color-coding of samples is based on the STRUCTURE program clustering from K1 to K4 (see Additional file 18: Table S11). The five isolates that were sequenced are marked by a closed circle. Label colors correspond to the genetic cluster the isolate belongs to based on STRUCTURE analysis. Some admixed samples were grouped together with samples from other populations. (B) Graphical presentation of the estimated membership coefficient, Q, for each of the 82 unique haplotypes in each of four genetic clusters (K). The most likely value of K inferred by STRUCTURE was 4. Each sample is shown as a horizontal line; the colored segments represent the proportion of the Q in each of the four ancestral genetic clusters. Individuals within each cluster are arranged according to the estimated cluster membership proportions (Q value). Detail of accessions in each cluster is provided in Additional file 18: Table S11. (PDF 437 KB) [file 12864_2014_6773_MOESM2_ESM.pdf]

**A**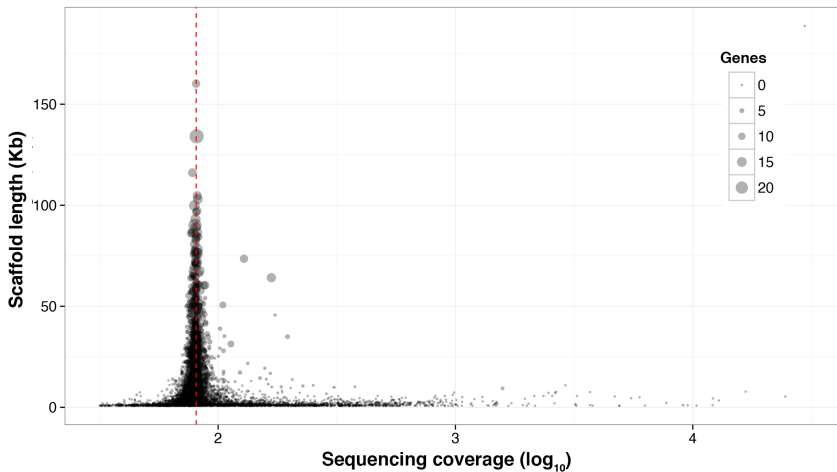**B**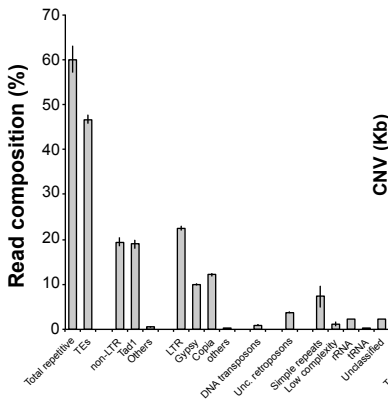**C**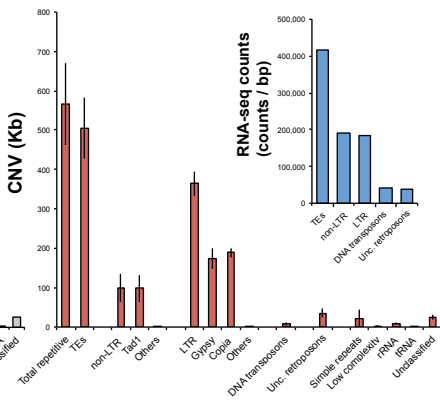**D**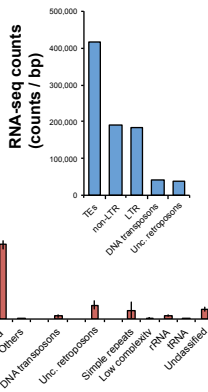

Supplement: Supplementary file 6 — Additional file 6: Figure S2: Analysis of the repetitive fraction of the E. necator genome. (A) Scatterplot showing the relation between scaffold length and sequencing coverage. The highly fragmented and gene-poor fraction of the assemblies shows higher sequencing depth than the long and gene-rich contigs, whose coverage match the median (and expected) value (red dashed line; 76X). (B) Bar plot showing the proportion of sequencing reads that mapped on the repetitive fraction of the E. necator genome. (C) Bar plot showing the size of copy number variant loci encompassing the annotated repetitive regions. (D) Bar plot showing the total number of RNA-seq reads that mapped on the annotated repetitive regions. (PDF 736 KB) [file 12864_2014_6773_MOESM6_ESM.pdf]

**A**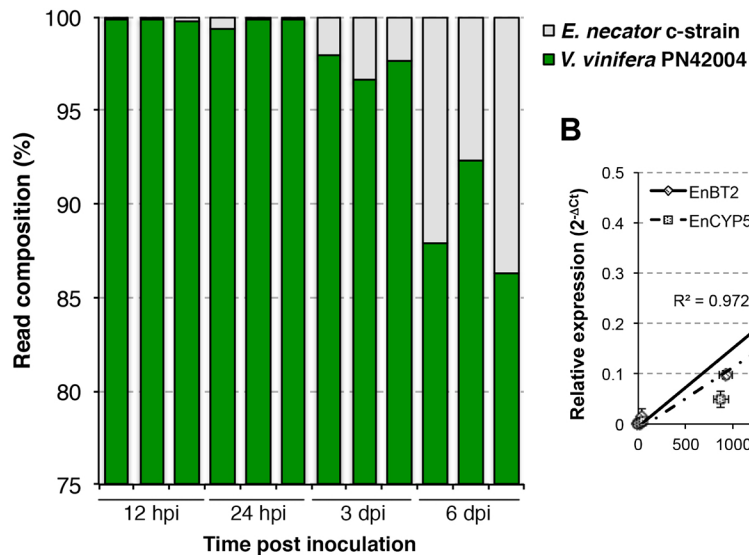**B**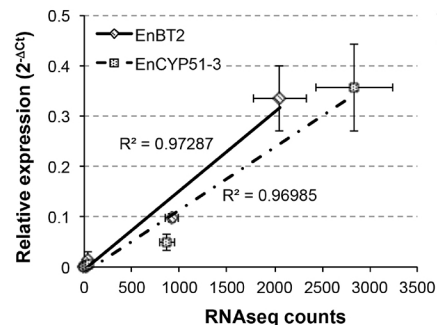**C**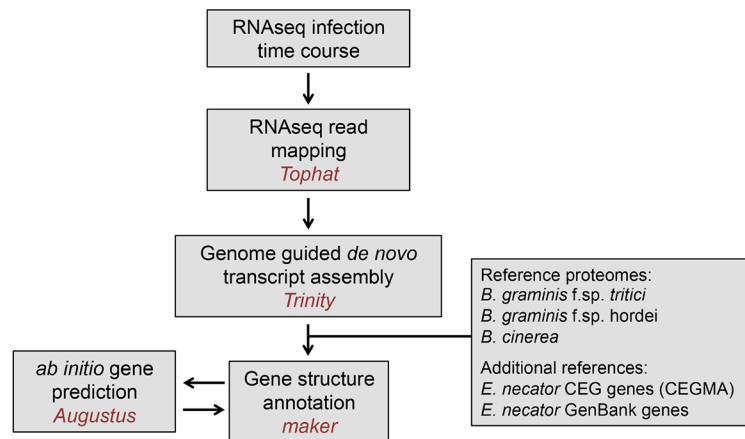**D**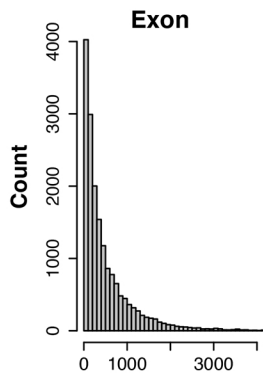**E**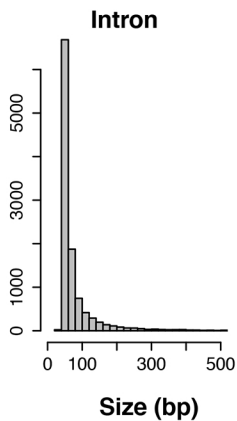**F**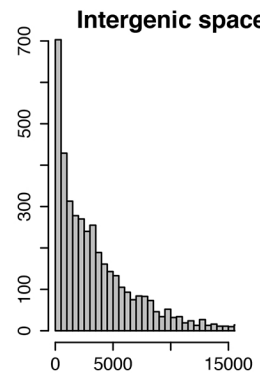**G**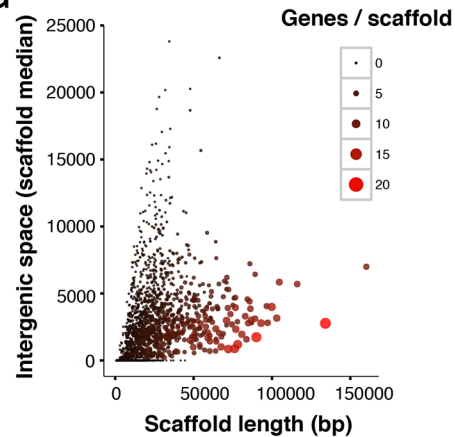**H**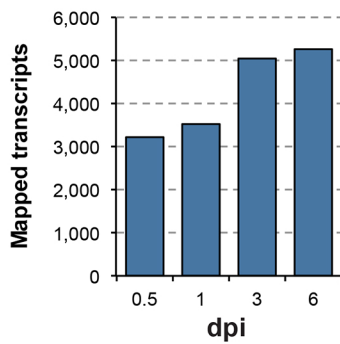**I**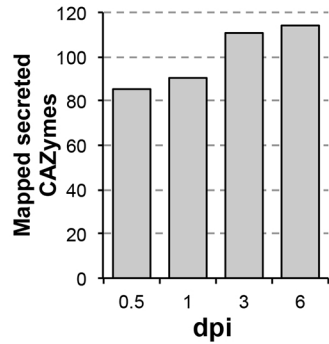**J**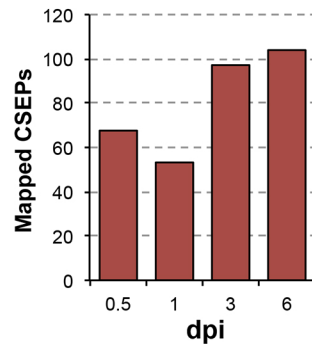

Supplement: Supplementary file 8 — Additional file 8: Figure S3: Transcriptome sequencing and gene prediction. (A) Proportion of V. vinifera and E. necator reads in the RNA-seq reads at each time point (hpi = hours post inoculation, dpi = days post inoculation). (B) Scatter plot showing the correlation between the expression of EnCYP51 and β-tubulin gene (EnBT) measured by RT-qPCR and the total E. necator RNA-seq reads. Expression values are presented as fold-EnCYP51 or EnBT (number of EnCYP51 molecules/number of VvACTIN molecules), calculated using the ΔCt method as described in [49]. (C) Diagram describing the pipeline used for gene prediction. (D-F) Histograms showing size distribution of exons ((D) mean size = 526.5 bp), introns ((E) mean size = 83.7 bp), and intergenic space ((F) mean size = 3,745.6 bp). (G) Scatter plot showing the relation between the median size of the intergenic space in each scaffold, scaffold length, and the number of genes per scaffold. Short and gene-poor scaffolds show the largest median intergenic space (top left), while long and gene-rich scaffolds (bottom right) show the smallest median intergenic space. (H-J) Bar graphs showing the total number of genes (H), CAZy genes (I), and CSEPs (J) mapped by the RNA-seq data during the infection time course. (PDF 3 MB) [file 12864_2014_6773_MOESM8_ESM.pdf]

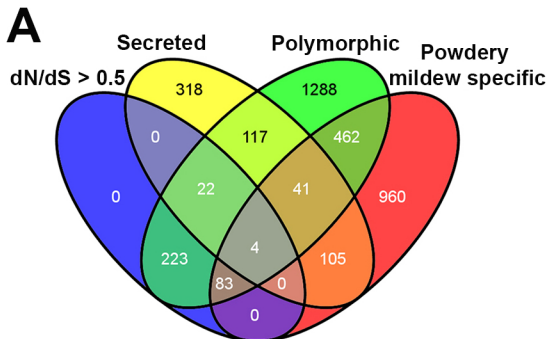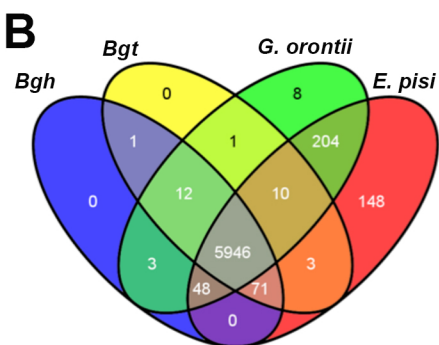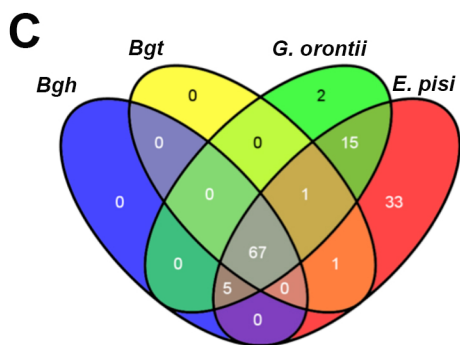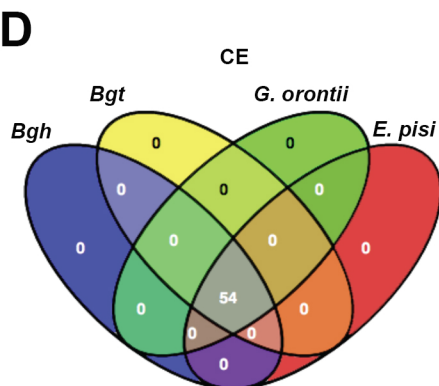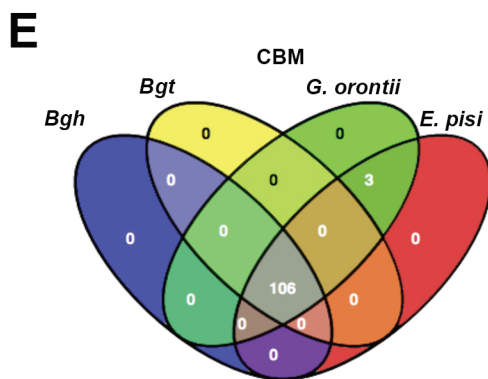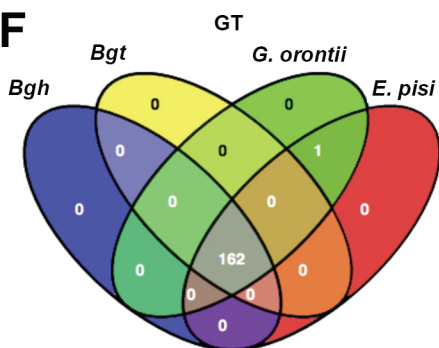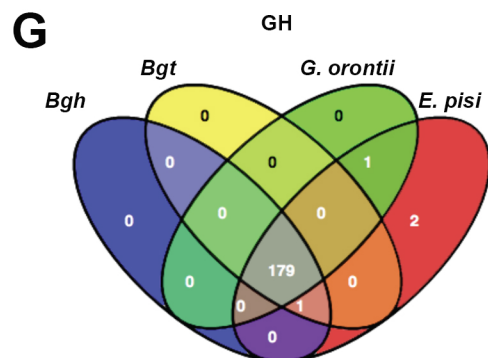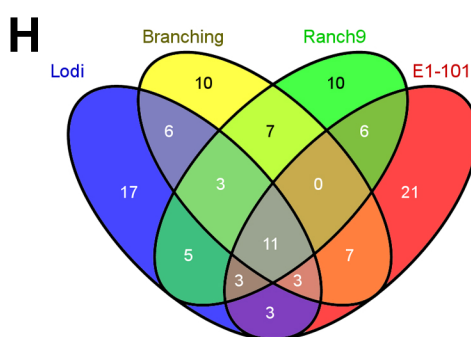

Supplement: Supplementary file 11 — Additional file 11: Figure S4: Characterization of the E. necator predicted proteome. (A) Venn diagram showing the overlap between predicted peptides with features associated with candidate effectors. Venn diagrams (B-G) showing the overlapping E. necator predicted peptides (B), CSEPs (C), and CAZymes (D-G; CEs = carbohydrate esterases; CBMs = carbohydrate binding modules; GTs = glycosyltransferases; GHs = glycosylhydrolases) matching the scaffolds of B. graminis f.sp. hordei (Bgh), B. graminis f.sp. tritici (Bgt), G. orontii, or E. pisi (TBLASTN, e-value < 10−3). (H) Venn diagram showing the number of unique or overlapping copy number variant protein-coding genes in the five sequenced E. necator isolates. (PDF 4 MB) [file 12864_2014_6773_MOESM11_ESM.pdf]

**A**

Biomass accumulation at 8 dpi estimated by qPCR  
(*EnEF1* Ct - *VvACTIN* Ct)

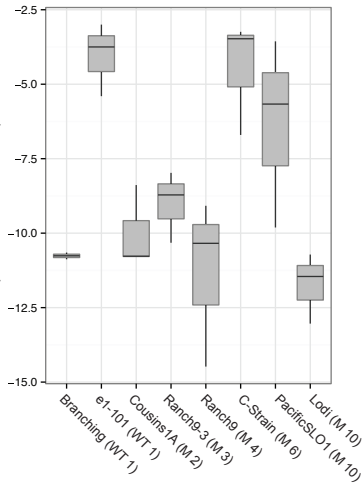**B**

Relative growth (%)

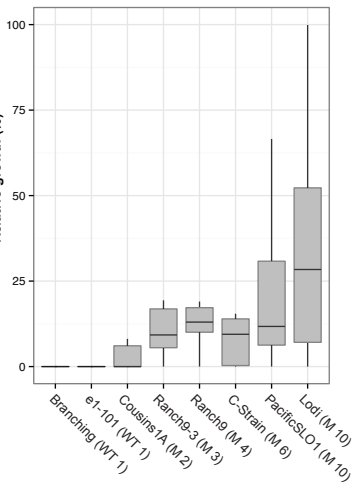

Supplement: Supplementary file 16 — Additional file 16: Figure S6: Fungicide resistance testing of E. necator isolates. Eight E. necator isolates were grown on leaf disks with and without DMI fungicide application (Rally® 40WSP). Results are an average of two separate trials, each with 3 independent replicates. Allelic form (WT = wild type, M = Y136F mutant) and EnCYP51 copy number estimated by qPCR are indicated in parentheses. (A) Box plot showing biomass accumulation in the absence of fungicide determined by qPCR amplification of the E. necator elongation factor EnEF1. (B) Boxplot showing the distribution of relative growth values. DNA accumulation levels were linearized with the formula 2 – (EnEF1 Ct – VvACT Ct) using the grape actin gene as reference. Growth results are reported as a percentage of the fungal biomass in the absence of fungicide (shown in (A)) to account for growth rate differences between the isolates in the absence of fungicide. (PDF 407 KB) [file 12864_2014_6773_MOESM16_ESM.pdf]
