# Supplementary material for: Adaptive genomic structural variation in the grape powdery mildew pathogen, Erysiphe necator
Source: BMC Genomics. 2014 Dec 9;15(1):1081. doi: 10.1186/1471-2164-15-1081 (PMC4298948; doi:10.1186/1471-2164-15-1081)
Supplement: Supplementary file 3 — Additional file 3: Text S1: Supplementary Material and Methods, Supplementary Results, Supplementary References. (PDF 153 KB) [file 12864_2014_6773_MOESM3_ESM.pdf]

Additional File 3: Text S1

Supplementary Materials for

Adaptive Genomic Structural Variation in the Grape Powdery Mildew Pathogen, *Erysiphe necator*

Laura Jones, Summaira Riaz, Abraham Morales-Cruz, Katherine C.H. Amrine, Brianna McGuire, W. Douglas Gubler, M. Andrew Walker, and Dario Cantu*

*Correspondence to: dacantu@ucdavis.edu

This PDF file includes:

Supplementary Text

- Supplementary Material and Methods
- Supplementary Results
- Supplementary References

Supplementary Material and Methods

SSR marker analysis. A total of 94 *E. necator* isolates collected between 2012 and 2013 from different locations in California, and four isolates from the Eastern United States (Additional File 1: Table S1) were used to generate fingerprint data. The four isolates from the Eastern United States were used as reference samples to assist in comparing microsatellite profiles obtained in different studies [1]. *E. necator* mycelia were harvested from three to four week-old colonies with sticky tape and DNA was extracted in 5% Chelex solution as described by [2]. Eleven previously published microsatellite markers were used to amplify the powdery mildew genomic DNA [1]. PCR was conducted in a total volume of 10 µL containing 5 ng genomic DNA and 1X Gold Buffer, 2 mM MgCl2, 0.8 mM of each dNTP, 0.13 units AmpliTaq Gold DNA polymerase, and 2 pmol of each primer (all from Applied Biosystems). Forward primers were labeled with one of three fluorescent dyes: 6-FAM, HEX, or NED. The thermal-cycler regime was 5 min at 94 °C, followed by 30 cycles of 30 s at 94 °C, 1 min at 56 °C, and 1 min at 72 °C, concluding with 1 cycle of 7 min at 72 °C. To generate microsatellite profiles, 0.5 to 0.8 µL of each of three amplified products were multiplexed using fluorescent dye and mixed with 10 µL formamide and 0.25 µL GeneScan 600 LIZ size standard (Applied Biosystems). Samples were denatured at 94 °C for 2 min prior to electrophoresis on 3500 Genetic Analyzer through a 50-cm capillary array with POP7 as the matrix. Allele binning, based on estimated size in base pairs (bp), and label editing were performed using GeneMapper 4.1 software (Applied Biosystems).

The fingerprint data set of 98 samples was analyzed with the program Microsatellite Toolkit [3] to identify the number of alleles, allele frequencies, and uniqueness of haplotypes. Simple matching distance (SMD [4]) was calculated with 11 SSR markers on all data sets. Tree clustering (unweighted-Neighbor) and Principal Coordinate Analysis (PCoA) were carried out with DARWIN software (version 5.0.158 [5]) to determine the number of groups. Model-based Bayesian analysis implemented in the software package STRUCTURE V2.3.1 (Additional File 18: Table S11; [6]) was used to infer the number of genetic clusters (K) with both data sets and to assign individuals to the most appropriate cluster. All simulations were run using the assumptions that haplotypes may have admixed ancestry and that allele frequencies are correlated [7]. Each run was implemented with a burn-in period of 100,000 steps followed by 250,000 Monte Carlo Markov Chain replicates. The most likely value for K was determined by calculating ΔK [8]. The results from STRUCTURE were displayed by DISTRUCT software [9].

Supplementary results

Population Structure of *E. necator* in California. Based on the fingerprint profile of 11 SSR markers, a total of 61 unique haplotypes were identified from a set of 94 samples that were purified from powdery mildew infected leaves or fruit

from different grape growing regions in California (Additional File 1: Table S1). SSR allele frequency data for the Type-A and Type-B isolates were obtained from [1]. In [1], Type-A *E. necator* isolates were monomorphic for all 11 SSR markers that were used in this study. Type-B isolates were polymorphic with two alleles at four markers and monomorphic for seven others, thus giving 16 possible allelic combinations with the 11 SSR markers. In order to compare the two data sets, four Eastern United States cross-reference samples (common to both studies) were used to convert the allele sizes. Three datasets of unique haplotypes were prepared: dataset 1 - synthetic data of both Type-A and Type-B isolates, California samples, and four reference samples from the Eastern United States; dataset 2 - California samples and four reference samples from the Eastern United States; dataset 3 - California samples only. All SSR markers were polymorphic with two to nine alleles for California isolates with the exception of marker EnMS9 that was monomorphic with one allele (Additional File 19: Table S12; Additional File 20: Table S13). On average, a total of four alleles were observed for California isolates. Private alleles were observed for four markers (EnMS1, EnMS4, EnMS10 and EnMS110) in California isolates that were not reported in earlier studies (Additional File 20: Table S13). The average number of the alleles was six for the combined dataset of 82 samples (17 Type-A and B isolates, four reference samples, and 61 unique California haplotypes).

Clustering analysis (un-weighted Neighbor Joining tree and Principle Coordinate analysis) was carried out separately on the combined dataset of 82 samples, and on 61 unique haplotypes from California with and without four Eastern United States reference samples. All analyses revealed two unique groups of *E. necator* within California that were distinct from the Eastern United States reference samples as well as Type-A and Type-B isolates. There were three isolates from California that grouped closely with the Eastern United States samples (Additional File 2: Figure S1A). Based on the estimated log probability of the data (ln Pr(X/K), [6]) and on ΔK [8], the most likely number of genetic clusters in the combined set of 82 unique haplotypes was four (Additional File 2: Figure S1B) and three when Type-A and B isolates were removed from the study set. When 82 samples were arranged according to their estimated degree of membership (Q) in each of the four clusters, an interesting picture emerged. Sixteen samples of European Type-B *E. necator* made one clear defined cluster. California samples formed two well-defined clusters, but clearly contained isolates of admix ancestry, mostly within each other and in some cases to the cluster of Eastern United States samples (Additional File 18: Table S11). Among the isolates that were sequenced, e1-101 was admixture of all four groups, C-strain was admixture of both California groups, and the other three isolates belonged to either California group with $Q > 0.98$ (Additional File 18: Table S11). Type-A samples were admixture of both California groups and Type-B. Three of the California samples with $Q > 0.90$ grouped with the reference samples. This was an unexpected result that indicates that *E. necator* in California has potentially greater genotypic diversity than previously hypothesized and most likely there are introductions from the Eastern United States. The other important result was that none of the California isolates showed strong affinity to the

European Type-B isolates. This result does not support the hypothesis based on the sequence comparisons of three nuclear gene regions of 146 isolates [2] that powdery mildew populations in California were recently introduced from Europe. In this study, there were only two samples that showed affinity with Q > 0.30 to the cluster of Type-B isolates. These results open up the question on the origins of Type-B powdery mildew and on the *E. necator* population structure in the United States. The cluster analysis was also carried out utilizing the Y136F mutation and CNV information. SSR marker allelic data did not show correlation with the CNV in *EnCYP51* and presence of the mutant allele (Y136F). These results indicate that the acquisition of multiple copies of *EnCYP51* gene has happened multiple times under selection pressure due to fungicide sprays.

Missing genes in the *E. necator* genome. Many fungi produce an array of secondary metabolites with important biological functions, such as stress protection and pathogenicity factors [10]. The four main groups of enzymes involved in secondary metabolite production in fungi are polyketide synthases (PKS), nonribosomal peptide synthases (NRPS), dimethylallyl tryptophan synthases (DMATS), and terpene synthases (TS). Similar to other obligate biotrophs, *E. necator* had a marked reduction in enzymes involved in secondary metabolism when compared to non-biotrophs [11-15]. We found only two nonribosomal peptide sythetases (NPRS) and one polyketide synthase (PKS). Genes encoding terpene cyclases and dimethylallyl tryptophan synthases were altogether absent as observed in *Bgh* [14].

In order to determine the extent of gene loss in the *E. necator* genome in comparison to the other sequenced powdery mildew pathogens, as described in [14] we identified a core set of 3,267 genes (core Ascomycete genes) that are present in the *Botrytis cinerea*, *Sclerotinia sclerotiorum*, *Magnaporthe oryzae*, and *Colletotrichum higginsianum* genomes (TBLASTN, e-value < $1e^{-10}$). Homologous sequences to the core Ascomycete genes were then searched in *E. necator* and the other powdery mildew genomes (*Bgh*, *Bgt*, *Erysiphe pisi*, and *Golovinomyces orontii*; TBLASTN (e-value < $1e^{-5}$)). Ninety five percent of the 153 core Ascomycete genes missing in *E. necator* were also missing in at least one of the other powdery mildew genomes (Additional File 21: Table S14). As reported in previous analyses of powdery mildews [14, 16] and other obligate biotrophs [11, 17, 18], *E. necator* showed a loss of enzymes involved in nitrate and sulfate metabolism, further supporting the convergent adaptation of obligate biotrophy [19].

Fungi have evolved a variety of mechanisms to limit and control TE activity, such as quelling [20], Meiotic Silencing of Unpaired DNA (MSUD; [21]), and Repeat Induced Point Mutation (RIP; [22]). RIP refers to C to T substitutions in repeated sequences after cytosine methylation. A single RIP passage can result in up to 30% of the GC base pairs in duplicated sequences becoming mutated to AT pairs. While DNA methylation provides short-term transcriptional silencing, the consequent increase in the mutation rate at the methylated sites permanently disables transposition activity. Elevated rates of transition mutations of cytosine in repetitive regions have been observed in many fungal species [23]. The genes

involved in these pathways are generally highly conserved throughout the Ascomycetes [24]. As observed in *Bgh* [14], we did not find any of the genes known to be required for RIP, while all genes involved in quelling and MSUD were identified. We did not find signatures of elevated GC to AT mutations in repeat-rich regions, which also suggests that the RIP pathway is not functional in *E. necator*, and may partly explain the proliferation of TEs and consequent genome size expansion.

Putative secreted carbohydrate active enzymes (CAZymes). Among the predicted 607 secreted peptides, we identified 120 Carbohydrate-Active enZYmes (CAZymes). CAZymes are families of enzymes involved in the breakdown, biosynthesis, and modification of carbohydrates and glycoconjugates. The CAZymes produced by fungal pathogens play an important role in the breakdown of plant cell wall and in host-pathogen interactions. The genomes and proteomes of obligately biotrophic fungi have been shown to have fewer CAZymes compared to hemibiotrophic and necrotrophic fungi. Similar to other biotrophs [13, 14, 25, 26], the genome of *E. necator* had reduced numbers of cell wall degrading enzymes (CWDE) from the CAZy carbohydrate esterase (CE), glycoside hydrolase (GH), and polysaccharide lyase (PL) functional classes. Like *Bgh*, *E. necator* lacked all CAZymes from PL classes. A homology search (TBLASTN, e-value < $1e^{-3}$) of *E. necator* CAZy genes with all powdery mildew scaffolds indicated that 99% of the total 530 CAZy genes (secreted and non-secreted) of *E. necator* match scaffolds in the other mildew species (Additional File 10: Table S6; Additional File 11: Figure S4D-G). Most of the CAZy genes were detected throughout the infection time course (Additional File 8: Figure S3I).

Candidate secreted effector proteins (CSEPs). Previous studies on powdery mildew pathogens focused on candidate secreted effector proteins (CSEPs) that lacked obvious BLAST hits outside of the powdery mildews [14, 15, 27]. Using the same criterion, we identified 150 CSEPs in *E. necator*, 26 of which were specific to *E. necator* (TBLASTN; e-value < $1e^{-3}$; Additional File 14: Table S9). Sixty-seven CSEPs were also present in all other sequenced powdery mildew genomes (TBLASTN; e-value < $1e^{-3}$; Additional File 14: Table S9). Among all the powdery mildew genomes, the largest number of homologous sequences to *E. necator* CSEPs was found in the genome of *E. pisi* (Additional File 11: Figure S4C). Importantly, the expression of 45% of the *E. necator* CSEPs was already detected at 0.5 dpi, suggesting a role for these proteins during very early stages of interaction with the host (Additional File 8: Figure S3J). Thirty-five of the 150 *E. necator* CSEPs contained the *N*-terminal [Y/F/W]xC motif which is associated with secreted proteins of haustoria-forming pathogenic fungi, including *Bgh* [28]. The observed frequency (23%) of [Y/F/W]xC motif in the predicted CSEP represents a significant enrichment (P=6.3 x 10^{-06}; one-sided Fisher's Exact Test) compared to the 10% frequency in the overall predicted *E. necator* proteome. As observed in *Bgh*, the most common (65%) amino acid of the motif is a tyrosine (YxC). Ninety-one percent of the CSEPs containing an *N*-terminal [Y/F/W]xC motif were also found in the *E. pisi* genome (TBLASTN; e-value < $1e^{-3}$), including

9 proteins with structural homology to microbial secreted ribonucleases, similar to prior observations in *Bgh* [29].

Effectors and other secreted proteins often show enhanced levels of positive selection compared to the rest of the proteome [17, 30]. To test for enrichment of non-synonymous mutations in *E. necator* CSEPs, we calculated the rates of synonymous (dS) and non-synonymous substitutions (dN) per synonymous site for secreted and non-secreted proteins, defining dN values > 0 as polymorphic and those with dN/dS ≥ 1 as being under diversifying selection. In contrast to *Bgh* and *Bgt*, which both showed an increased in CSEP dN and dN/dS compared to non-secreted proteins [15, 16], *E. necator* did not show an increase in polymorphisms or positive selection in candidate effectors. Of the 150 CSEPs, only 46 proteins (30.7%) were polymorphic (dN > 0) and just 1 protein showed signature of positive selection (dN/dS value ≥ 1), while the non-secreted proteins (5,926 total) were 34.7% dN > 0 and 1.2% dN/dS ≥ 1.

Supplementary References

1. Frenkel O, Portillo I, Brewer MT, Péros JP, Cadle-Davidson L, Milgroom MG: **Development of microsatellite markers from the transcriptome of Erysiphe necator for analysing population structure in North America and Europe**. *Plant Pathol* 2012, **61**(1):106-119.
2. Brewer MT, Milgroom MG: **Phylogeography and population structure of the grape powdery mildew fungus, *Erysiphe necator*, from diverse *Vitis* species**. *BMC Evol Biol* 2010, **10**:268.
3. Park S: **Trypanotolerance in West African cattle and the population genetic effects of selection**. University of Dublin; 2001.
4. Bowcock AM, Ruiz-Linares A, Tomfohrde J, Minch E, Kidd JR, Cavalli-Sforza LL: **High resolution of human evolutionary trees with polymorphic microsatellites**. *Nature* 1994, **368**(6470):455-457.
5. Perrier X, Jacquemoud-Collet JP (2006): **DARwin software** [http://darwin.cirad.fr/]
6. Pritchard JK, Stephens M, Donnelly P: **Inference of population structure using multilocus genotype data**. *Genetics* 2000, **155**(2):945-959.
7. Falush D, Stephens M, Pritchard JK: **Inference of population structure using multilocus genotype data: linked loci and correlated allele frequencies**. *Genetics* 2003, **164**(4):1567-1587.
8. Evanno G, Regnaut S, Goudet J: **Detecting the number of clusters of individuals using the software STRUCTURE: a simulation study**. *Mol Ecol* 2005, **14**(8):2611-2620.
9. Rosenberg NA: **DISTRUCT: a program for the graphical display of population structure**. *Mol Ecol Notes* 2004, **4**:137-138.
10. Keller NP, Turner G, Bennett JW: **Fungal secondary metabolism - from biochemistry to genomics**. *Nat Rev Microbiol* 2005, **3**(12):937-947.
11. Baxter L, Tripathy S, Ishaque N, Boot N, Cabral A, Kemen E, Thines M, Ah-Fong A, Anderson R, Badejoko W, Bittner-Eddy P, Boore JL, Chibucos MC, Coates M, Dehal P, Delehaunty K, Dong S, Downton P, Dumas B, Fabro G, Fronick C, Fuerstenberg SI, Fulton L, Gaulin E, Govers F, Hughes L, Humphray S, Jiang RH, Judelson H, Kamoun S, *et al*: **Signatures of adaptation to obligate**

biotrophy in the *Hyaloperonospora arabidopsidis* genome. *Science* 2010, **330**(6010):1549-1551.
12. Collemare J, Griffiths S, Iida Y, Karimi Jashni M, Battaglia E, Cox RJ, de Wit PJ: **Secondary metabolism and biotrophic lifestyle in the tomato pathogen *Cladosporium fulvum***. *PLoS One* 2014, **9**(1):e85877.
13. O'Connell RJ, Thon MR, Hacquard S, Amyotte SG, Kleemann J, Torres MF, Damm U, Buiate EA, Epstein L, Alkan N, Altmuller J, Alvarado-Balderrama L, Bauser CA, Becker C, Birren BW, Chen Z, Choi J, Crouch JA, Duvick JP, Farman MA, Gan P, Heiman D, Henrissat B, Howard RJ, Kabbage M, Koch C, Kracher B, Kubo Y, Law AD, Lebrun MH*, et al*: **Lifestyle transitions in plant pathogenic *Colletotrichum* fungi deciphered by genome and transcriptome analyses**. *Nat Genet* 2012, **44**(9):1060-1065.
14. Spanu PD, Abbott JC, Amselem J, Burgis TA, Soanes DM, Stuber K, Ver Loren van Themaat E, Brown JK, Butcher SA, Gurr SJ, Lebrun MH, Ridout CJ, Schulze-Lefert P, Talbot NJ, Ahmadinejad N, Ametz C, Barton GR, Benjdia M, Bidzinski P, Bindschedler LV, Both M, Brewer MT, Cadle-Davidson L, Cadle-Davidson MM, Collemare J, Cramer R, Frenkel O, Godfrey D, Harriman J, Hoede C*, et al*: **Genome expansion and gene loss in powdery mildew fungi reveal tradeoffs in extreme parasitism**. *Science* 2010, **330**(6010):1543-1546.
15. Wicker T, Oberhaensli S, Parlange F, Buchmann JP, Shatalina M, Roffler S, Ben-David R, Dolezel J, Simkova H, Schulze-Lefert P, Spanu PD, Bruggmann R, Amselem J, Quesneville H, Ver Loren van Themaat E, Paape T, Shimizu KK, Keller B: **The wheat powdery mildew genome shows the unique evolution of an obligate biotroph**. *Nat Genet* 2013, **45**(9):1092-1096.
16. Hacquard S, Kracher B, Maekawa T, Vernaldi S, Schulze-Lefert P, Ver Loren van Themaat E: **Mosaic genome structure of the barley powdery mildew pathogen and conservation of transcriptional programs in divergent hosts**. *Proc Natl Acad Sci U S A* 2013, **110**(24):E2219-E2228.
17. Kemen E, Gardiner A, Schultz-Larsen T, Kemen AC, Balmuth AL, Robert-Seilaniantz A, Bailey K, Holub E, Studholme DJ, Maclean D, Jones JD: **Gene gain and loss during evolution of obligate parasitism in the white rust pathogen of *Arabidopsis thaliana***. *PLoS Biol* 2011, **9**(7):e1001094.
18. Duplessis S, Cuomo CA, Lin Y-c, Aerts A, Tisserant E, Grabherr MG, Kodira CD, Kohler A, Kües U, Lindquist EA, Lucas SM, Grigoriev IV, Szabo LJ, Martin F: **Obligate biotrophy features unraveled by the genomic analysis of rust fungi**. *Proc Natl Acad Sci U S A* 2011, **108**(22):9166-9171.
19. Spanu PD: **The genomics of obligate (and nonobligate) biotrophs**. *Annu Rev Phytopathol* 2012, **50**:91-109.
20. Romano N, Macino G: **Quelling: transient inactivation of gene expression in *Neurospora crassa* by transformation with homologous sequences**. *Mol Microbiol* 1992, **6**(22):3343-3353.
21. Shiu PKT, Raju NB, Zickler D, Metzenberg RL: **Meiotic silencing by unpaired DNA**. *Cell* 2001, **107**(7):905-916.
22. Cambareri E, Singer M, Selker E: **Recurrence of repeat-induced point mutation (RIP) in *Neurospora crassa***. *Genetics* 1991, **127**(4):699-710.
23. Clutterbuck AJ: **Genomic evidence of repeat-induced point mutation (RIP) in filamentous ascomycetes**. *Fungal Genet Biol* 2011, **48**(3):306-326.
24. Nakayashiki H, Kadotani N, Mayama S: **Evolution and diversification of RNA silencing proteins in fungi**. *J Mol Evol* 2006, **63**(1):127-135.

25. Duplessis S, Spanu PD, Schirawski J: **Biotrophic fungi (powdery mildews, rusts, and smuts)**. In *The ecological genomics of fungi.* Edited by Martin F. Hoboken, NJ: John Wiley & Sons, Inc.; 2014:149-168.
26. Zhao Z, Liu H, Wang C, Xu J-R: **Comparative analysis of fungal genomes reveals different plant cell wall degrading capacity in fungi**. *BMC Genomics* 2013, **14**:274.
27. Pedersen C, van Themaat EVL, Mcguffin LJ, Abbott JC, Burgis TA, Barton G, Bindschedler LV, Lu X, Maekawa T, Weßling R, Cramer R, Thordal-christensen H, Panstruga R, Spanu PD: **Structure and evolution of barley powdery mildew effector candidates**. *BMC Genomics* 2012, **13**:694.
28. Godfrey D, Bohlenius H, Pedersen C, Zhang Z, Emmersen J, Thordal-Christensen H: **Powdery mildew fungal effector candidates share N-terminal Y/F/WxC-motif**. *BMC Genomics* 2010, **11**:317.
29. Pliego C, Nowara D, Bonciani G, Gheorghe DM, Xu R, Surana P, Whigham E, Nettleton D, Bogdanove AJ, Wise RP, Schweizer P, Bindschedler LV, Spanu PD: **Host-induced gene silencing in barley powdery mildew reveals a class of ribonuclease-like effectors**. *Mol Plant Microbe Interact* 2013, **26**(6):633-642.
30. Kamoun S: **A catalogue of the effector secretome of plant pathogenic oomycetes**. *Annu Rev Phytopathol* 2006, **44**:41-60.
