# Supplementary material for: Adaptive genomic structural variation in the grape powdery mildew pathogen, Erysiphe necator
Source: BMC Genomics. 2014 Dec 9;15(1):1081. doi: 10.1186/1471-2164-15-1081 (PMC4298948; doi:10.1186/1471-2164-15-1081)
Supplement: Supplementary file 15 — Additional file 15: Figure S5: Presence of the EnCYP51 mutant allele (Y136F) conferring DMI resistance in isolates collected from fungicide-treated vineyards. The three isolates collected from fungicide-sprayed vineyards (C-strain, Lodi, and Ranch9) showed multiple copies of EnCYP51 with all copies containing the Y136F mutation, while the two isolates from vineyards not treated with fungicides (e1-101 and Branching) each contained a singly copy of the wild type, fungicide susceptible EnCYP51 allele. (A) Diagram of sequencing read coverage and the presence of Y136F mutation. Boxed solid red line represents the presence of only one allele at the 136 position of EnCYP51. Multicolored lines represent positions with mixed alleles in other regions. (B) Sanger sequencing chromatogram of EnCYP51. Single peaks at the 136 position confirm that all duplicated copies are in the same allelic form. All 94 California isolates were genotyped as described in [16] and allelic form were validated by Sanger sequencing. (C) Protein alignment of EnCYP51 shows that the Y136F substitution was the only non-synonymous polymorphism detected across the isolates. (PDF 2 MB) [file 12864_2014_6773_MOESM15_ESM.pdf]

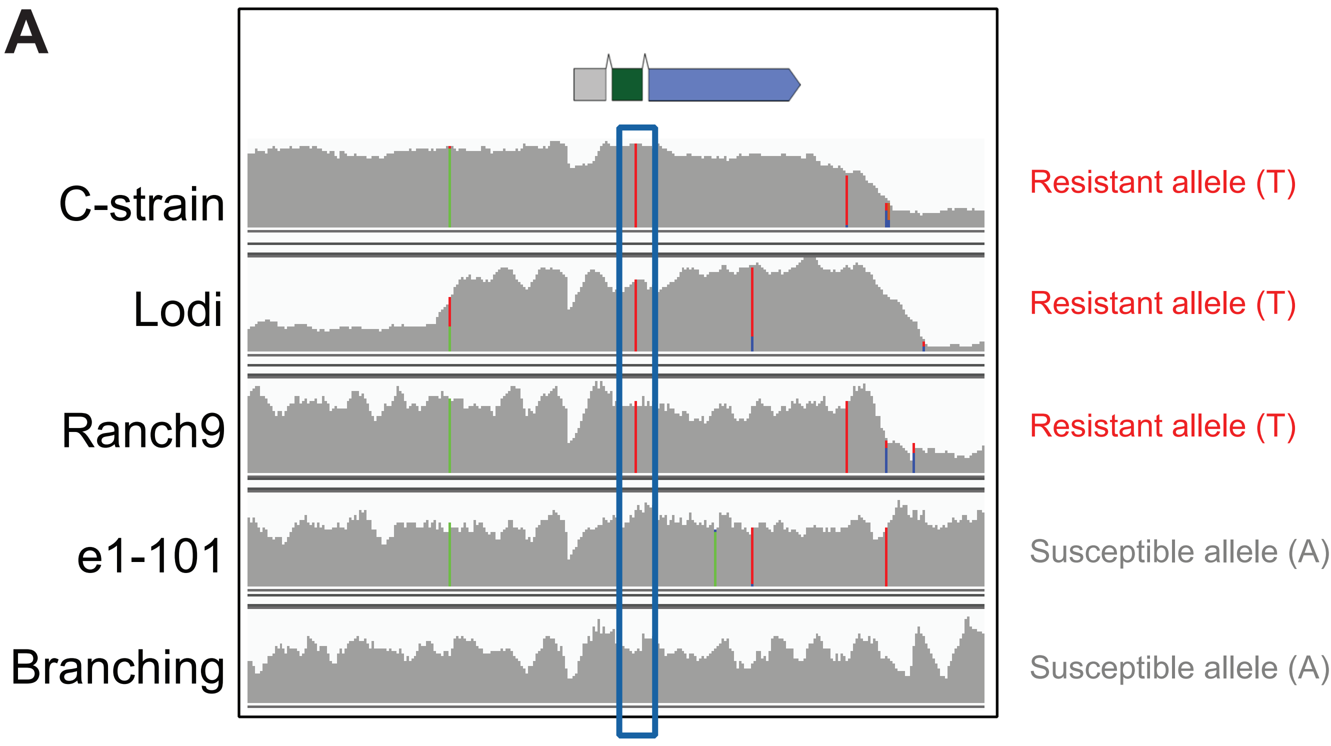

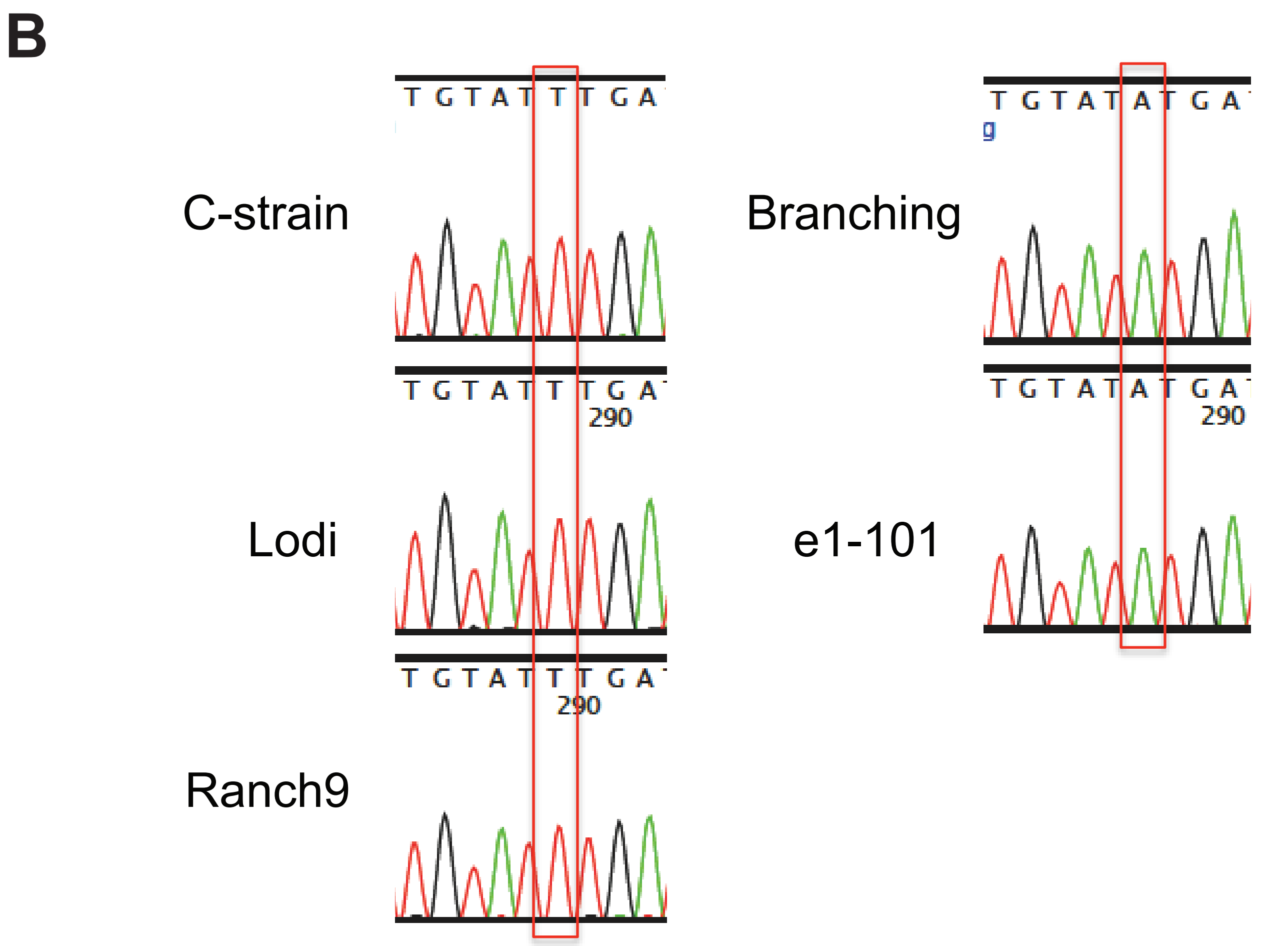

C

```
C-strain   MYIADILSDLLTQQTTRYGWIFMVTSIAFSIILLAVGLNVLSQLLFRRPYEPPVVFHWFP 60
Lodi       MYIADILSDLLTQQTTRYGWIFMVTSIAFSIILLAVGLNVLSQLLFRRPYEPPVVFHWFP 60
Ranch9     MYIADILSDLLTQQTTRYGWIFMVTSIAFSIILLAVGLNVLSQLLFRRPYEPPVVFHWFP 60
e1-101     MYIADILSDLLTQQTTRYGWIFMVTSIAFSIILLAVGLNVLSQLLFRRPYEPPVVFHWFP 60
Branching  MYIADILSDLLTQQTTRYGWIFMVTSIAFSIILLAVGLNVLSQLLFRRPYEPPVVFHWFP 60
           ************************************************************

C-strain   IIGSTISYGIDPYKFYFDCRAKYGDIFTFILLGKKVTVYLGLQGNNFILNGKLKDVNAEE 120
Lodi       IIGSTISYGIDPYKFYFDCRAKYGDIFTFILLGKKVTVYLGLQGNNFILNGKLKDVNAEE 120
Ranch9     IIGSTISYGIDPYKFYFDCRAKYGDIFTFILLGKKVTVYLGLQGNNFILNGKLKDVNAEE 120
e1-101     IIGSTISYGIDPYKFYFDCRAKYGDIFTFILLGKKVTVYLGLQGNNFILNGKLKDVNAEE 120
Branching  IIGSTISYGIDPYKFYFDCRAKYGDIFTFILLGKKVTVYLGLQGNNFILNGKLKDVNAEE 120
           ************************************************************

C-strain   IYTNLTTPVFGRDVVFDCPNSKLMEQKKFMKTALTIEAFHSYVTIIQNEVEAYINNCVSF 180
Lodi       IYTNLTTPVFGRDVVFDCPNSKLMEQKKFMKTALTIEAFHSYVTIIQNEVEAYINNCVSF 180
Ranch9     IYTNLTTPVFGRDVVFDCPNSKLMEQKKFMKTALTIEAFHSYVTIIQNEVEAYINNCVSF 180
e1-101     IYTNLTTPVFGRDVVYDCPNSKLMEQKKFMKTALTIEAFHSYVTIIQNEVEAYINNCVSF 180
Branching  IYTNLTTPVFGRDVVYDCPNSKLMEQKKFMKTALTIEAFHSYVTIIQNEVEAYINNCVSF 180
           ***************:********************************************

C-strain   QGESGTVNISKVMAEITIYTASHALQGEEVRENFDSSFAALYHDLDMGFTPINFTFYWAP 240
Lodi       QGESGTVNISKVMAEITIYTASHALQGEEVRENFDSSFAALYHDLDMGFTPINFTFYWAP 240
Ranch9     QGESGTVNISKVMAEITIYTASHALQGEEVRENFDSSFAALYHDLDMGFTPINFTFYWAP 240
e1-101     QGESGTVNISKVMAEITIYTASHALQGEEVRENFDSSFAALYHDLDMGFTPINFTFYWAP 240
Branching  QGESGTVNISKVMAEITIYTASHALQGEEVRENFDSSFAALYHDLDMGFTPINFTFYWAP 240
           ************************************************************

C-strain   LPWNRARDHAQRTVARTYMNIIQARREEKRSGENKHDIMWELMRSTYKDGTPVPDREIAH 300
Lodi       LPWNRARDHAQRTVARTYMNIIQARREEKRSGENKHDIMWELMRSTYKDGTPVPDREIAH 300
Ranch9     LPWNRARDHAQRTVARTYMNIIQARREEKRSGENKHDIMWELMRSTYKDGTPVPDREIAH 300
e1-101     LPWNRARDHAQRTVARTYMNIIQARREEKRSGENKHDIMWELMRSTYKDGTPVPDREIAH 300
Branching  LPWNRARDHAQRTVARTYMNIIQARREEKRSGENKHDIMWELMRSTYKDGTPVPDREIAH 300
           ************************************************************

C-strain   MMIALLMAGQHSSSSTSSWIMLWLAARPDIMEELYEEQLRIFGSEKPFPPLQYEDLSKLQ 360
Lodi       MMIALLMAGQHSSSSTSSWIMLWLAARPDIMEELYEEQLRIFGSEKPFPPLQYEDLSKLQ 360
Ranch9     MMIALLMAGQHSSSSTSSWIMLWLAARPDIMEELYEEQLRIFGSEKPFPPLQYEDLSKLQ 360
e1-101     MMIALLMAGQHSSSSTSSWIMLWLAARPDIMEELYEEQLRIFGSEKPFPPLQYEDLSKLQ 360
Branching  MMIALLMAGQHSSSSTSSWIMLWLAARPDIMEELYEEQLRIFGSEKPFPPLQYEDLSKLQ 360
           ************************************************************

C-strain   LHQNVLKEVLRLHAPIHSIMRKVKNPMIVPGTKYVIPTSHVLISSPGCTSQDATFFPDPL 420
Lodi       LHQNVLKEVLRLHAPIHSIMRKVKNPMIVPGTKYVIPTSHVLISSPGCTSQDATFFPDPL 420
Ranch9     LHQNVLKEVLRLHAPIHSIMRKVKNPMIVPGTKYVIPTSHVLISSPGCTSQDATFFPDPL 420
e1-101     LHQNVLKEVLRLHAPIHSIMRKVKNPMIVPGTKYVIPTSHVLISSPGCTSQDATFFPDPL 420
Branching  LHQNVLKEVLRLHAPIHSIMRKVKNPMIVPGTKYVIPTSHVLISSPGCTSQDATFFPDPL 420
           ************************************************************

C-strain   KWDPHRWDIGSGKVLGNDAVDEKYDYGYGLTSTGASSPYLPFGAGRHRCIGEQFATLQLV 480
Lodi       KWDPHRWDIGSGKVLGNDAVDEKYDYGYGLTSTGASSPYLPFGAGRHRCIGEQFATLQLV 480
Ranch9     KWDPHRWDIGSGKVLGNDAVDEKYDYGYGLTSTGASSPYLPFGAGRHRCIGEQFATLQLV 480
e1-101     KWDPHRWDIGSGKVLGNDAVDEKYDYGYGLTSTGASSPYLPFGAGRHRCIGEQFATLQLV 480
Branching  KWDPHRWDIGSGKVLGNDAVDEKYDYGYGLTSTGASSPYLPFGAGRHRCIGEQFATLQLV 480
           ************************************************************

C-strain   TIMATMVRFFRFRNIDGKQGVVKTDYSSLFSMPLAPALIGWEKR 523
Lodi       TIMATMVRFFRFRNIDGKQGVVKTDYSSLFSMPLAPALIGWEKR 523
Ranch9     TIMATMVRFFRFRNIDGKQGVVKTDYSSLFSMPLAPALIGWEKR 523
e1-101     TIMATMVRFFRFRNIDGKQGVVKTDYSSLFSMPLAPALIGWEKR 523
Branching  TIMATMVRFFRFRNIDGKQGVVKTDYSSLFSMPLAPALIGWEKR 523
           ********************************************
```
